# Sleep pattern differences in women at midlife: a large-scale multi-country smartwatch study (SONAR)

**DOI:** 10.1093/sleep/zsag197

**Published:** 2026-07-28

**Authors:** Claudio N Soares, Carina Dinkel-Keuthage, Fiona C Baker, MinSoeng Kim, JungWon Lee, Julie Dorey, Ann-Kathrin Frenz, DongHyun Lee, Cecilia Caetano, Carsten Moeller, Nils Schoof, Frank Kramer, Elaina Bolinger, Marcelo Graziano Custodio, Ricky Choi, Jennifer Kanady, Pauline M Maki

**Affiliations:** Department of Psychiatry, Queen’s University, Kingston, ON, Canada; Real-World Evidence Centre of Excellence, Bayer AG, Berlin, Germany; Center for Health Sciences, SRI International, Menlo Park, CA, United States; MX Division, Health Platform Development Group, Samsung Electronics, Suwon, Korea; MX Division, Health Platform Development Group, Samsung Electronics, Suwon, Korea; Real-World Evidence Centre of Excellence, Bayer Healthcare SAS, La Garenne-Colombes, France; Medical Affairs Statistics, Bayer AG, Wuppertal, Germany; MX Division, Health AI Algorithm Lab., Samsung Electronics, Suwon, Korea; Medical Evidence Women's Healthcare, Bayer Consumer Care, Basel, Switzerland; Real-World Evidence Centre of Excellence, Bayer AG, Berlin, Germany; Medical Evidence Women's Healthcare, Bayer AG, Berlin, Germany; R&D Data Science & AI, Bayer AG, Wuppertal, Germany; R&D Data Science & AI, Bayer AG, Berlin, Germany; Medical Evidence Women's Healthcare, Bayer Consumer Care, Basel, Switzerland; Digital Health, Samsung Electronics America, California, United States; Digital Health, Samsung Electronics America, California, United States; Department of Psychiatry, Psychology and Obstetrics and Gynecology, University of Illinois, Chicago, ON, United States

**Keywords:** sleep, age, midlife, wearable, women

## Abstract

**Study Objectives:**

To evaluate associations between age and objectively measured sleep in women.

**Materials and Methods:**

We analyzed ~3.8 million nights’ data from 192 500 female Galaxy Smartwatch users aged 20–65 years (United States, South Korea, Germany, France, and the United Kingdom) with at least seven nights’ sleep data during March 2024. We evaluated associations between age and total sleep period duration (TSPD), total sleep time (TST), and wakefulness after sleep onset (WASO; log-transformed) using linear mixed models, adjusting for repeated user measures, country, body mass index, weekday/weekend night, and daily activity time. Reference group was age 20–24 years. Separate models were run for a comparison group of men.

**Results:**

In women WASO increased notably at midlife; average increases of +5 mins (+15%) for 50–54 years, +9 minutes (+25%) for 55–59 years, and +11 minutes (+32%) for 60–64 years vs. reference group (corresponding increases in men were +3 minutes [+9%], +6 minutes [+16%], and +9 minutes [+27%]). Compared with the reference group, age differences in TSPD and TST were ≤5 minutes less and ≤17 minutes less, respectively. Effects of age on TST and WASO were similar across Western countries; in South Korea, TSPD and TST were shorter across all ages, while increases in WASO were apparent from early adulthood.

**Conclusions:**

A pronounced increase in WASO was seen in women at midlife. Women in this stage of life could benefit from early identification and tailored interventions to improve their sleep as part of overall health counseling.

## Graphical Abstract

**Graphical abstract:**
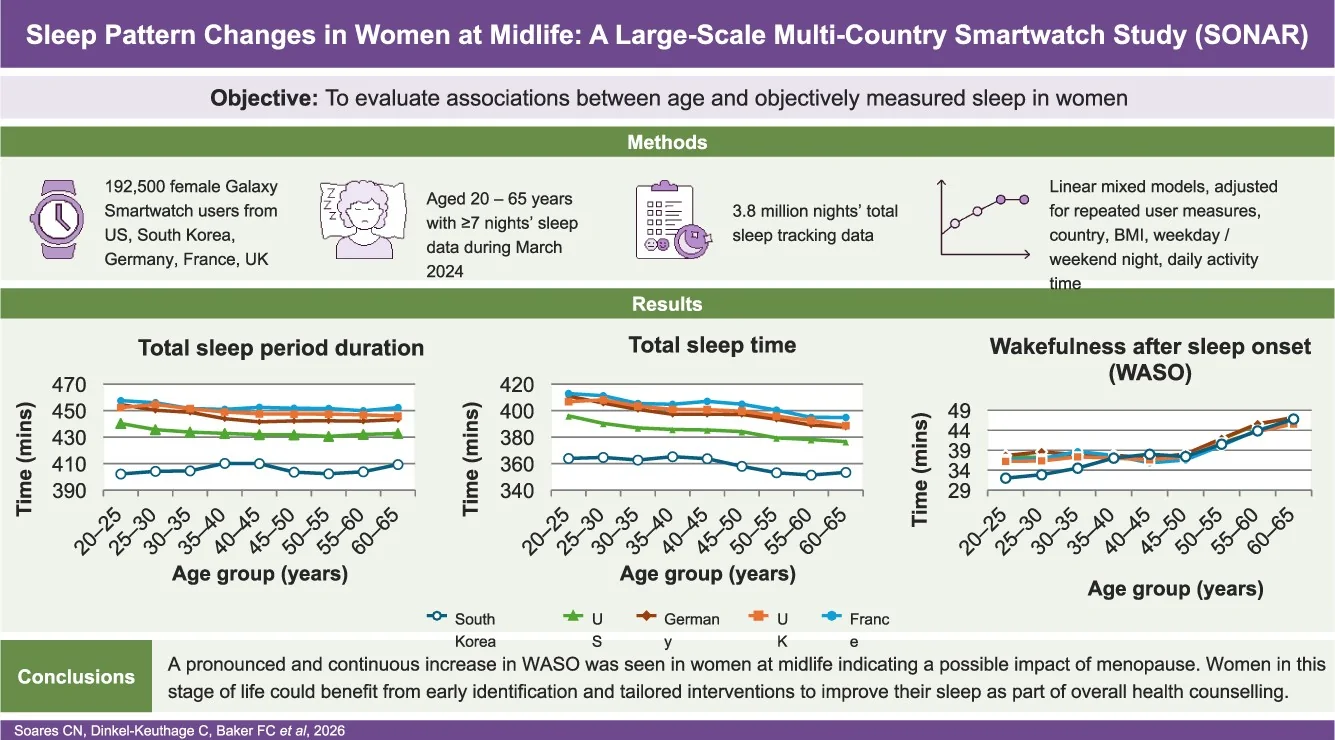

**Statement of Significance**

In this analysis of ~3.8 million nights’ data from 192 500 female users of the Galaxy Smartwatch (United States, South Korea, Germany, France, and the United Kingdom), a notable increase in duration of night-time wakefulness was seen at midlife (50–64 years). The increase in wakefulness after sleep onset was more pronounced than that seen in men, suggesting a sex difference in sleep patterns at midlife. Further research is needed to examine menopause and other factors that may differentially contribute to awakenings. Wearable technologies provide large-scale, real-world objective data to advance research, and offer an opportunity for women to track their sleep, facilitating discussions with healthcare practitioners and guiding a personalized approach to symptom management.

## Introduction

Sleep patterns change over the adult lifespan [1–5] and there is some evidence to suggest that certain sleep dimensions, such as sleep efficiency and the frequency of night-time awakenings, differ between sexes, especially around midlife [3, 4, 6–8]. It is during this period of life—around the late 40s to early 50s—that most women transition to menopause [6]. Sleep disturbances associated with menopause occur in 40%–60% of this population [7, 8], and are characterized by frequent night-time awakening and increased time awake after sleep onset [7]. These sleep symptoms can lead to irritability and fatigue [9], and are associated with cognitive dysfunction [7], reduced quality of life and mental well-being, and lower work productivity [10, 11], as well as increased cardiometabolic risk [12] and healthcare utilization [11, 13].

Aside from menopause, other factors that can affect sleep include sociodemographic factors such as age and sex [14], physical activity [15], stress and mental health problems [16–18], pain and physical illness [17–19], obesity [20], alcohol consumption [21], certain medications [22], and shift work [23]. There can also be weekend and seasonal effects, with night-time sleep duration shorter during weekdays than weekends [1, 20, 24, 25], and shorter in summer than winter months [26–28]. Furthermore, there is some evidence for geographical, perhaps cultural, differences in sleep duration that persist across age and sex, with shorter overall sleep duration in Asian vs. Western countries [1, 29].

Wearable digital health technologies (DHTs), such as smartwatches and rings, provide the opportunity to capture objectively measured sleep data over time in large-scale real-world populations. Furthermore, objective data on sleep can complement important subjective information captured with self-reported outcomes. Accordingly, we conducted a large-scale multi-country smartwatch study using approximately 3.8 million nights’ data to evaluate associations between age and objectively measured sleep parameters in women. We also evaluated other factors that could potentially affect sleep in women across age groups, including body mass index (BMI), country of residence, and physical activity level. Furthermore, as our main interest was in sleep patterns during midlife—capturing the years that most women transition to menopause—we evaluated these associations in a comparison group of men.

## Materials and methods

### Data source and study design

SONAR was a cross-sectional study using objectively measured sleep data collected from Galaxy smartwatch users (up to Galaxy Watch series 6) from France, Germany, South Korea, the United Kingdom, and the United States. The Galaxy Watch series captures sleep measures such as total sleep time (TST) and awakenings using a 3-axial accelerometer sensor (to detect movement) and a photoplethysmography sensor (to derive pulse rate). Validation studies of the Galaxy Watch have demonstrated high sensitivity (95.4%) for sleep detection [30], and high specificity (91.0%–95.4%) for detecting wake [30, 31], based on polysomnography as the gold standard. The Galaxy Watch also captures data on daily step count and physical activity level (minutes of “active” time per day based on the sum of all recorded exercise sessions plus the total duration of periods with increased step counts), measures (or proxy measures) of which have been validated previously against gold-standard measures [32–34]. The watch also holds data on users’ self-entered height and weight. These data are held in Samsung Health’s consumer database (SamsungHealth).

### Study population

We obtained sleep tracking data from 192 500 female users of the Galaxy Watch (random samples of 38 500 women from each country*)* who were aged 20–65 years and had at least seven nights’ sleep data between 1st and 28th March 2024 (chosen to avoid seasonal effects) from the S-Health database. For inclusion, individuals were also required to have entered their height and weight and to have a BMI of ≥18.5 kg/m^2^, and a sleep duration between 3 and 14 hours that started within the timeframe of 8 p.m. to 8 a.m. (in line with standard filters for sleep duration used in previous research) [20, 35].

#### Comparison sample of men

Owing to our interest in sleep patterns in women around midlife, we obtained a comparison sample of male Galaxy Watch users to identify potential differences at this time of life between the sexes. A total of 145 000 men were selected based on a random sample of 29 000 from each country (the sample size being determined by the country with the smallest available sample, in this case France), contributing a total 2 806 501 nights’ sleep data. Inclusion criteria were the same as for women. Users identified as male with a birth date of 1 January 1990 were excluded due to this being the default setting for users who had not entered a birth date.

### Night-time sleep parameters

For each individual, we calculated the average value for three sleep parameters: total sleep period duration (TSPD), defined as the number of minutes between sleep onset and final awakening; TST, defined as the total minutes asleep within the TSPD; and wakefulness after sleep onset (WASO), defined as the number of minutes spent awake after having initially fallen asleep and before final awakening (awake time between individual sleep sessions of the same night was also accounted for). To improve data quality, outliers in wake stage duration between two sleep episodes above the median + 1.5 × IQR (160 min) were excluded. Further details can be found in the Supplement.

### Age and covariates

Age, according to individual user profile, was categorized as 20–24 years, 25–29 years, 30–34 years, 35–39 years, 40–44 years, 45–49 years, 50–54 years, 55–59 years, and 60–65 years. BMI was categorized as 18.5–24.9 kg/m^2^ (healthy weight), 25.0–29.9 kg/m^2^ (overweight), and ≥ 30 kg/m^2^ (obesity). Level of tracked activity was categorized by number of steps per day (average over the month): sedentary (<5000 per day), low activity (5000–7499 per day), somewhat active (7500–9999 per day), active (10000–12 499 per day), and highly active (>12 500 per day). Physical activity time was categorized into tertiles of minutes of active time per day (average over the month) and categorized with tertiles on the basis that there are currently no published categories for wearable physical activity. Weekday night was considered as Sunday to Thursday nights, and weekend nights were Friday and Saturday nights.

### Statistical analysis

Each of the three sleep parameters were summarized by age category using mean with *SD*, and median with first and third quartiles (Q1, Q3). Linear mixed effect models were used to explore associations between sleep parameters and age (5-year age groups) as well as with country, BMI category, physical activity (step counts and active time tertiles), and weekday/weekend, controlling for user (repeated measures) and covariates. Reference categories were 20–25 years for age, the United States for country, healthy weight (BMI of 18.5–24.9 kg/m^2^), weekday for weekend/weekday, lower tertile for daily active time, and sedentary for daily step count category. Separate models were run for TSPD, TST, and WASO (log-transformed to account for non-normality)—three models in total, each exploring associations with age and the other covariates. Additionally, we explored potential age–country interactions by fitting an interaction term in the model between age and country. Separate models were run for the comparison group of men.

## Results

### Study population

A total of 3 797 367 nights’ sleep data were analyzed from the 192 500 women in the study sample (average 20 nights per user, range 7–28). Characteristics of these women are shown in Table 1. Approximately a third of women (30.6%) were aged ≥50 years, and age distributions of women and the comparator group of men were comparable across countries (Figures S1; Tables S1 and S2). Average BMI was 27.6 kg/m^2^, and 29.2% of women were obese (Table S3). Women undertook an average 6817 steps/day and were physically active for an average 76 minutes/day (Table S3). Average values for TSPD, TST, and WASO for women across age groups are shown in Table S4 (and in Table S5 for males). Among midlife women, the average TSPD was 7 hours, 18 minutes for age 45–49 years, 7 hours, 17 minutes for age 50–54 years, and 7 hours, 18 minutes for age 55–59 years. Corresponding values for average TST were 6 hours, 32 minutes; 6 hours, 27 minutes; and 6 hours, 24 minutes, respectively, and for WASO were 47 minutes, 50 minutes, and 54 minutes, respectively (descriptive data on the number of awakenings can be found in Table S6). Among women aged 20–24 years (the reference group in the regression models), average values were 7 hours, 19 minutes for TSPD, 6 hours, 37 minutes for TST, and 42 minutes for WASO. Findings from the regression models for associations between age (and other covariates) and sleep parameters are shown in Figure 1 (TSPD), Figure 2 (TST), and Figure 3 (WASO) and are described hereafter.

**Table 1:** Characteristics of the 192 500 female Galaxy smartwatch users used in the analysis

	Female Galaxy smartwatch users *N* = 192 500
**Age, years**	
20–24	6607 (3.4)
25–29	14 705 (7.6)
30–34	22 019 (11.4)
35–39	30 545 (15.9)
40–44	31 742 (16.5)
45–49	27 845 (14.5)
50–54	24 435 (12.7)
55–59	20 108 (10.4)
60–65	14 494 (7.5)
**BMI, kg/m** ^**2**^	
Mean (SD)	27.6 (10.6)
18.5–24.9 (healthy weight), n (%)	82 673 (42.9)
25.0–29.9 (overweight), n (%)	53 636 (27.9)
≥30 (obesity), n (%)	56 191 (29.2)
**Daily step count**	
Mean (SD)	6817.2 (3281.0)
<5000 (sedentary)	64 182 (33.3)
5000–7499 (low active)	64 691 (33.6)
7500–9999 (somewhat active)	37 930 (19.7)
10 000–12 499 (active)	16 180 (8.4)
>12 500 (highly active)	9517 (4.9)
**Physical activity time, minutes**	
Mean (*SD*)	75.7 (36.0)
First tertile	57.5
Second tertile	85.2
Min	0.3
Max	1182.2
Lower tertile (average active time ≤ first tertile)	69 140 (35.9)
Mid tertile (average active time > first tertile and ≤ second tertile)	63 185 (32.8)
Upper tertile (average active time > second tertile)	60 175 (31.3)

BMI, body mass index; *SD*, standard deviation

**Figure 1:**
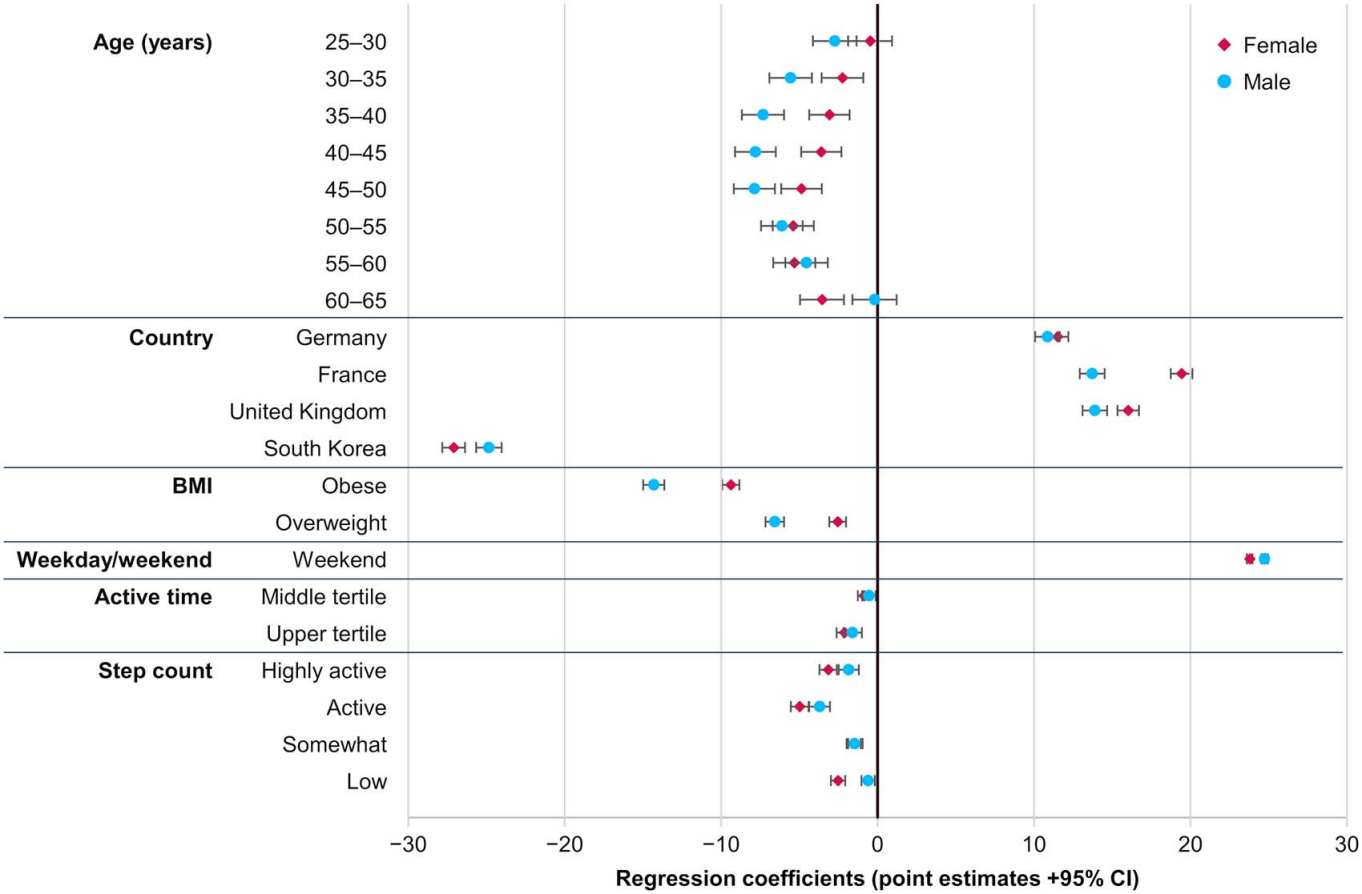
Age, country, BMI, physical activity, and step count category differences in TSPD compared with the respective reference category (linear mixed effect regression models) among females (pink triangle estimates) and males (blue circle estimates). Analysis was performed on data from 192 500 female and 145 000 male galaxy watch users. Note: Estimates for males and females in South Korea were highly similar. Reference categories were 20.0–24.9 years for age, the United States for country, healthy weight (18.5–24.9 kg/m^2^) for BMI, weekday for weekend/weekday, lower tertile for daily active time, and sedentary for daily step count category. BMI, body mass index; TSPD, total sleep period duration; US, United States.

**Figure 2:**
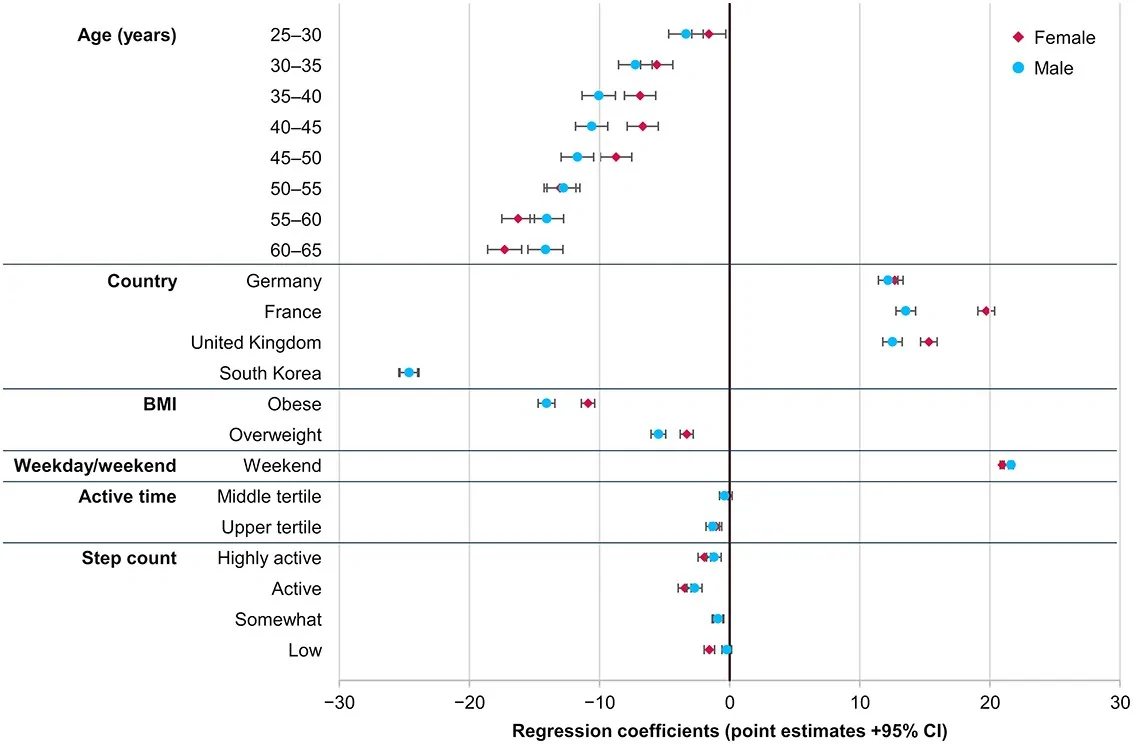
Age, country, BMI, physical activity, and step count category differences in TST compared with the respective reference category (linear mixed effect regression models) among females (pink triangle estimates) and males (blue circle estimates). Analysis was performed on data from 192 500 female and 145 000 male galaxy watch users. Reference categories were (20.0–24.9 years for age, the United States for country, healthy weight (18.5–24.9 kg/m^2^) for BMI, weekday for weekend/weekday, lower tertile for daily active time, and sedentary for daily step count category. BMI, body mass index; TST, total sleep time; US, United States.

**Figure 3:**
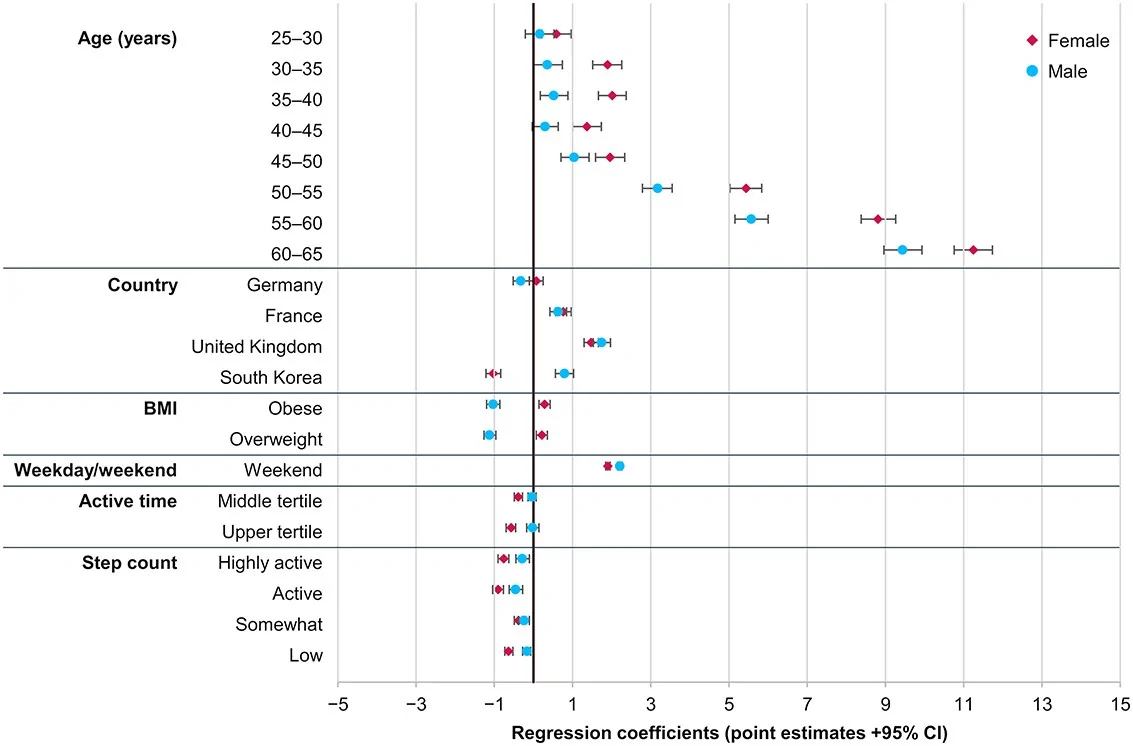
Age, country, BMI, physical activity, and step count category differences in WASO compared with the respective reference category (linear mixed effect regression models) among females (pink triangle estimates) and males (blue circle estimates). Analysis was performed on data from 192 500 female and 145 000 male galaxy watch users. Reference categories were 20.0–24.9 years for age, the United States for country, healthy weight (18.5–24.9 kg/m^2^) for BMI, weekday for weekend/weekday, lower tertile for daily active time, and sedentary for daily step count category. BMI, body mass index; US, United States; WASO, wakefulness after sleep.

### Associations with age

#### Total sleep period duration

TSPD showed a slight *U*-shaped distribution, decreasing up to age 50–59 years, and then slightly increasing at age 60–65 years (Figure 1, Table S7). Compared with women aged 20–24 years (reference group), average TSPD was shortest among midlife women, being an average 5 minutes shorter in the 45–49 years, 50–54 years, and 55–59 years age groups (Figure 1, Table S7). In the corresponding analysis among men, average TSPD was shortest among the 40–45 years and 45–49 years age groups (average 8 minutes shorter in both groups vs. age 20–24 years) (Figure 1, Table S8).

#### Total sleep time

TST declined with age. Compared to women age 20–24 years, TST was notably shorter in women aged 50–54 years (13.0 minutes less [−3.3%]) and those aged 60–65 years (17.3 minutes less [−4.4%]) (Figure 2, Table S7). In contrast, men showed a steadier decrease in TST with age and was 14.2 minutes shorter (−3.8%) for men aged 60–65 years vs. 20–24 years (Figure 2, Table S8).

#### Wakefulness after sleep onset

Generally, WASO either remained similar or increased slightly up to midlife where a jump was seen at age 50–54 years. Compared to women aged 20–24 years, average increases in WASO were: 5.4 minutes more (+15.3%) for 50–54 years, 8.8 minutes more (+24.9%) for 55–59 years, and 11.2 minutes more (+31.7%) for 60–65 years. Corresponding average increases in WASO for males were: 3.2 minutes more (+9.2%) for 50–54 years, 5.6 minutes more (+16.1%) for 55–59 years, and 9.4 minutes more (+27.0%) for 60–65 years. In women, the highest increase between consecutive age groups was from 45–49 to 50–54 years (+3.5 minutes, 9.3%). In contrast, for men, the highest increase between consecutive age groups was from 55–59 years to 60–64 years (+3.9 minutes, 9.6%).

### Associations with country, weekends, BMI, and activity level

#### Total sleep period duration

In the regression model, notable differences in TSPD were seen between countries. Compared with women from the US, TSPD was shorter in those from South Korea (an average 27.1 minutes shorter) and longer in those from Germany, France and the United Kingdom (an average 11.5, 19.4, and 16.0 minutes longer, respectively) (Figure 1, Table S7). Weekend effects were also notable, being an average 23.8 minutes longer on weekend nights vs. weekday nights (Figure 1, Table S7). Being obese or overweight was associated with shorter TSPD relative to having a healthy weight (an average 2.6 and 9.4 minutes less, respectively), and tracked activity (step count)/physical activity time differences in TSPD were <5.0 minutes (Figure 1, Table S7). Similar patterns were seen among men (Figure 1, Table S8).

#### Total sleep time

Similar to TSPD, clear country-level differences in TST were seen in the regression model, with shorter duration among women in South Korea vs. the United States (average 24.7 minutes shorter), and longer duration in women from Germany, France, and the United Kingdom vs. the United States (an average 12.7, 19.7, and 15.3 minutes longer, respectively) (Figure 2, Table S7). Weekend nights were also associated with longer TST (20.9 minutes longer) vs. weekday nights. (Figure 2, Table S7). Women who were overweight or obese had shorter TST vs. those with a healthy weight (an average 3.3 and 10.9 minutes less, respectively) (Figure 2, Table S7). Tracked activity (step count)/physical activity-related differences in TST were small (<3.5 minutes). Similar patterns were observed among men (Figure 2, Table S8).

#### Wakefulness after sleep onset

In contrast to TSPD and TST, country differences in WASO were small, with average duration only minimally longer in women from Europe (max. 1.5 minutes longer), and minimally shorter in those from South Korea (1 minute shorter) vs. the United States (Figure 3, Table S7). A weekend effect on WASO was also small (1.9 minutes longer for weekend vs. weekday nights), and only minimal effects on WASO were seen with BMI (maximum 0.3 minutes longer for overweight/obesity vs. healthy weight) and tracked activity (step count)/physical activity level (<1 minute difference) (Figure 3, Table S7). Similar patterns were observed in men.

### Age-country interactions

Findings from the regression models including an interaction term between age and country are shown in Figure 4.

**Figure 4:**
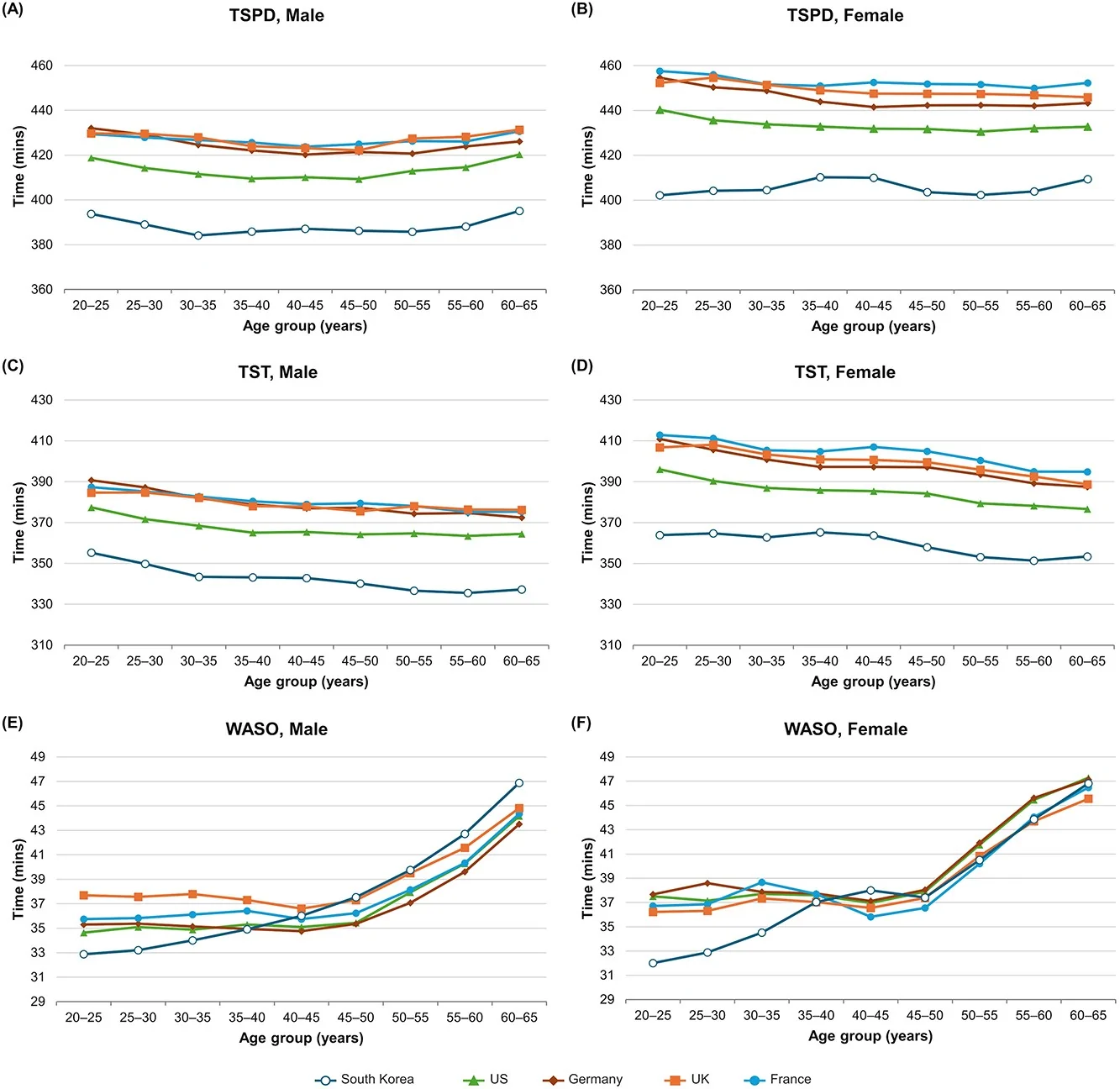
Mean expected durations by age and country in females and males for (A) TSPD, (B) TST, and (C) WASO. Estimates were derived from linear mixed effect regression models with an interaction term between age group and country and with BMI, weekday/weekend, activity level and step count kept at the reference category). Analysis was performed on data from 192 500 female and 145 000 male galaxy watch users. Reference categories were healthy weight (18.5–24.9 kg/m^2^) for BMI, weekday for weekend/weekday, lower tertile for daily active time, and sedentary for daily step count category. BMI, body mass index; TSPD, total sleep period duration; TST, total sleep time; UK, United Kingdom; US, United States.

#### Total sleep period duration and TST

Age-related differences in TSPD and TST were consistent across Western countries yet differed from women in South Korea (*p* < .01). In the latter, TSPD increased slightly from age 30–34 years to 40–44 years, then decreased slightly at midlife before increasing again at age 60–65 years (Figure 4A**)**, whereas TST was consistent between age 20–24 years and 40–44 years, before decreasing until age 55–59 years and increasing at age 60–64 years **(**Figure 4C). In males, age-related changes in TSPD and TST were generally consistent across all countries (Figure 4B and 4D).

#### Wakefulness after sleep onset

Age-related increases in WASO starting around midlife were generally consistent across Western countries. In South Korea, WASO was shorter among women aged 20–34 years relative to those of similar age in Western countries, although it increased with age with a notable increase from age 30–34 years (*p* < .05; Figure 4E); country-differences were no longer apparent upon reaching age 35–40 years. In men, WASO patterns were more similar between countries, although the increase in WASO in men from South Korea was evident from an earlier age (35–39 years; *p* < .05) (Figure 4F).

## Discussion

Our large-scale multi-country study of objectively measured night-time sleep patterns in women of different age groups from the United States, South Korea, Germany, France, and the United Kingdom, provide valuable information on age-related differences in three critical sleep measures as well as inter-country differences. Based on around 3.8 million nights’ sleep data from close to 200 000 Galaxy smartwatch users, we observed clear age-related increases in WASO in women at midlife, as well as age-related decreases in TSPD and TST, after adjusting for country, BMI, physical activity, and weekday/weekend effects.

Although we also observed an age-related increase in WASO at midlife in men, this was more modest than in women. Notable increases in WASO at midlife, particularly in women, have been reported previously in studies of objectively [20, 36] or subjectively [3, 37] measured sleep. In a global study of over 11 million nights’ wearable device sleep data, Jonasdottir *et al.* [20] observed a sharper increase in the proportion of women with WASO in the midlife years (after age 40–44 years) compared to men. Similarly, in a large-scale study of body acceleration data collected from a chest-worn accelerometer in over 68 000 individuals from Japan, Li *et al* [36] found that WASO was higher for women in their 50s, relative to levels for those in their 20s, whereas for men, significant increases in WASO became apparent only in the 60s. One could hypothesize the possible effects of the menopausal transition on sleep patterns in women during the midlife years. However, our findings based on a large-scale wearable dataset do require further corroboration from studies in which associations between menopause staging/symptoms specifically and WASO could be more directly explored, beyond aging-related changes. Furthermore, several other changes that commonly occur in women during midlife could contribute to the observed difference in WASO between the sexes. Increased stress from more demanding caregiving responsibilities [38], and a higher prevalence of mood symptoms and disorders [39] among women are also plausible contributory factors, with mood symptoms worsening in the menopausal transition and the association between mood and sleep disturbance being bidirectional [40]. Changes in body fat deposition and distribution at midlife (also associated with menopause in women) could also be a contributing factor if this has differential effects on sleep between the sexes.

Thus far, findings from small polysomnography studies relating to differences in WASO between pre-menopause and peri-/post-menopause life stages have been mixed [41–45], whereas self-reported sleep data have been more consistent in finding poorer sleep quality during peri- and post-menopause relative to pre-menopause [46]. Additionally, a large cross-sectional survey from the United States found that, compared to an elderly reference group, the odds of self-reported sleep disturbances were highest at midlife (ages 45–54 years) in women, an effect not seen in men [37]. However, among 300 women who reached menopause in the United States longitudinal Study of Women’s Health Across the Nation, WASO became shorter between ages 52 years and 64 years in fully-adjusted models based on a combination of actigraphy and survey data [42]. Sleep disturbances are among the most disruptive of symptoms in midlife women, and can be associated with menopause [7, 8], either related to, or independent of, vasomotor symptoms [10, 47]. Menopausal sleep disturbances have been associated with impaired menopause-specific and general health-related quality of life, reduced work productivity, and the ability to perform daily activities [48]. Consumer wearable DHTs provide the opportunity for women to continuously track their sleep, for example across midlife, thereby increasing awareness about sleep patterns and the potential effects of other bothersome symptoms such as vasomotor symptoms. In turn, these data could help facilitate discussions with healthcare practitioners, guide a personalized approach to symptom management, and support treatment adherence and monitoring. To help women manage these symptoms during midlife, it is also important that healthcare practitioners, including menopause counselors, proactively ask about sleep. Furthermore, devices such as the Galaxy Smartwatch not only enable the generation of considerably large real-world datasets for research purposes but can also be used in clinical trials and practice to generate additional insights into sleep quality.

Although no formal analysis was undertaken to compare sleep parameters between the sexes, on average, women had longer TSPD and TST than men across age groups, consistent with findings from other large studies of objectively [1, 20] or self-reported sleep [3]. On the other hand, women tended to have greater WASO than men across age groups. Of note, polysomnography studies have tended to show that men have more WASO and poorer sleep efficiency than women across a wide age range, with sleep architecture being better preserved into old age in women, controlling for obstructive sleep apnea [45, 49]. While we found TST to decrease over age in both sexes, previous reports of sleep duration in relation to age have been mixed. Some studies have reported a decline in objectively / subjectively measured TST through to old age [1, 5], yet others have either reported a more U-shape curve with the nadir occurring in mid-life (40s and 50s) [36, 50] or have not evaluated sleep in the elderly [20]. The oldest age group in our study was 60–65 years, and TSPD was longer at this age than during the earlier midlife years, indicating a possible U-shape distribution with age in line with findings from Japan by Li *et al* [36]. Consistent with other large-scale analyses of wearable device captured sleep data, [1, 29] and survey data [51], we saw clear inter-country differences in TSPD and TST, being shorter among individuals from South Korea vs. Western countries across age groups. This observation, seen for both sexes, likely reflects socio-cultural differences that influence sleep, particularly longer working hours, different work cultures, but also differences in evening meal times, leisure activity, and the sleep environment [29], including wider adoption of co-sleeping with babies and young children in Asian vs. Western cultures [52, 53]. Similarly, although age-related differences in WASO were consistent between the United States and Europe, the pattern differed for women from South Korea. Younger women from South Korea had shorter WASO than those of equivalent ages from Western countries, perhaps indicating they have better sleep efficiency in the context of a short TSPD, whereas from their late 30s, WASO increased, and aligned with women from Western countries, despite continuing to have lower TSPD. We observed a pattern of longer TSPD and TST at weekends being 24 minutes and 21 minutes longer, respectively vs. weekdays), consistent with other DHTstudies [1, 20, 29, 54]. Women in our study slept on average 21 minutes longer at weekends vs weekdays, in line with findings of greater sleep duration at weekends (average 20–35 minutes longer) reported by Scott *et al* [54] among women using an FDA-cleared under-mattress sleep sensor. Similarly, in a smart wrist-band-based study, Jonasdottir *et al.* [20] found weekend–weekday differences in sleep duration of 25-40 minutes across the age range of 19–55 years, with a later sleep onset and offset on weekends. In our study, TSPD and TST were on average 9 minutes less, and 11 minutes less, respectively, among women who were obese vs. a healthy weight. Few other wearable studies have evaluated associations between BMI or activity levels and sleep. Jonasdottir *et al.* [20] similarly reported small effects of overweight and obesity in reduced sleep duration vs. underweight or normal weight women, while a meta-analysis of 33 studies on sleep-activity levels in adults, daytime physical activity was not significantly associated TST at the individual level [55]. For WASO, we only observed small associations with these factors, with the aforementioned age-effects being dominant.

### Limitations

Our study used cross-sectional data, rather than long-term, longitudinal sleep data as women age, and we did not include individuals aged over 65 years or under 20 years. Also, Galaxy Watch users with at least seven nights’ sleep data are unlikely to be representative of the general population of each respective country, particularly for factors that could affect sleep; for example, they could have been more health conscious with an interest in sleep, and/or wealthier. This limits the generalizability of our results to the respective general populations. Also, because we opted to use the average sleep parameters for users with at least seven nights’ sleep data, regardless of the number of nights’ data available, parameter estimates for some Galaxy Watch users might be more representative of their typical sleep compared to others. As the Galaxy Watch is not approved as a medical device, the data in this study were not of medical grade. Furthermore, validation studies of specific Galaxy Watch models suggest high sensitivity for sleep detection [30], high specificity for detecting wake [30, 31], strong validity in measuring steps [32], low mean absolute percentage error (MAPE) for measuring heart rate [33], and moderate MAPE for measuring energy expenditure [34]. There might have been some misclassification of sleep data due to the inability of the Galaxy Watch to distinguish between being asleep and being awake in bed, leading to an underestimation of WASO and an overestimation of TST [56]. Furthermore, wearables tend to have poor performance in people with more sleep disturbances [57, 58]. We also recognize that shorter sleep opportunity or duration may contribute to shorter WASO, and further research is needed to investigate this. We acknowledge some lack of age granularity due to the 5-year age categorization, and that misclassification of covariates could have occurred due to user recording errors or entering incorrect data for height/weight. As 88% Galaxy Watch users in the entire S-Health database have a BMI record recorded in the 6 months before our study period, BMI could have been misclassified for a small proportion of our study population. Lastly, other data on factors that can affect sleep were not available, including medications used to treat sleep problems or menopausal symptoms, menopausal stage, frequency and severity of vasomotor symptoms and other potentially bothersome symptoms in women, pregnancy, breast feeding, and comorbidities such as sleep apnea. However, our study was largely intended to be descriptive, and predictive or causal analyses are not intended.

## Conclusions

In this large-scale multi-country smartwatch study, clear increases in WASO were observed in women during midlife, and these changes were more pronounced than those seen in men at equivalent age. This suggests a possible impact of midlife changes on sleep in women. Identifying sleep disturbances during this stage of life can be aided by wearable DHTs, which enable women to track their sleep, facilitating discussions with healthcare practitioners and guiding a personalized approach to symptom management.

## Supplementary Material

Supplementary_material_zsag197

## Data Availability

The anonymized and privacy-protected data used in this study are property of Samsung Electronics and are not shared publicly.
