# Supplementary material for: Sleep pattern differences in women at midlife: a large-scale multi-country smartwatch study (SONAR)
Source: Sleep. 2026 Jul 28;49(10):zsag197. doi: 10.1093/sleep/zsag197 (PMC13658266; doi:10.1093/sleep/zsag197)
Supplement: Supplementary file 1 [file supplementary_material_zsag197.zip › SONAR_supplement_zsag197.docx]

SUPPLEMENT

# Sleep Pattern Differences in Women at Midlife: A Large-Scale Multi-Country Smartwatch Study (SONAR)

Claudio N Soares^1^, Carina Dinkel-Keuthage^2^, Fiona C. Baker^3^, Minsoeng Kim^4^, Jungwon Lee^4^, Julie Dorey^5^, Ann-Kathrin Frenz^6^, Donghyun Lee^4^, Cecilia Caetano^7^, Carsten Moeller^2^, Nils Schoof^2^, Frank Kramer^6^, Elaina Bolinger^2^, Marcelo Graziano Custodio^7^, Ricky Choi^8^, Jennifer Kanady^8^, Pauline M Maki^9^

1. Department of Psychiatry, Queen’s University, Kingston, Ontario, Canada
2. Bayer AG, Berlin, Germany
3. SRI International, Menlo Park, CA, USA
4. Samsung Electronics, Suwon, Korea
5. Bayer Healthcare SAS, La Garenne-Colombes, France
6. Bayer AG, Wuppertal, Germany
7. Bayer Consumer Care, Basel, Switzerland
8. Samsung Electronics America, California, USA
9. Department of Psychiatry, Psychology and Obstetrics & Gynecology, University of Illinois, Chicago, United States

**Author for correspondence:** Claudio N Soares, Department of Psychiatry, Queen’s University, Kingston, Ontario, Canada; email: [Claudio.Soares@kingstonhsc.ca](mailto:Claudio.Soares@kingstonhsc.ca)

## CONTENTS

[Supplementary Methods	3](#_Toc234999586)

[Figure S1. Age distribution of (A) females and (B) males by country.	4](#_Toc234999587)

[Table S1. Age distribution of females overall and by country.	5](#_Toc234999588)

[Table S2. Age distribution of males overall and by country.	6](#_Toc234999589)

[Table S3. BMI, and daily step count and physical activity time in women overall and by country.	7](#_Toc234999590)

[Table S4. Mean and median values for TSPD, TST, and WASO in women overall and by 5-year age category.	8](#_Toc234999591)

[Table S5. Mean and median values for TSPD, TST, and WASO in men overall and by 5-year age category.	9](#_Toc234999592)

[Table S6. Mean and median number of awakenings in women overall and by 5-year age category.	10](#_Toc234999593)

[Table S7. Estimate (95% CI) for difference in TSPD, TST, and WASO for age and other covariates compared with the respective reference group in females (regression model).	11](#_Toc234999594)

[Table S8. Estimate (95% CI) for difference in TSPD, TST, and WASO for age and other covariates compared with the respective reference group in men (regression model).	13](#_Toc234999595)

### Supplementary Methods

#### Preparation of sleep data prior to analysis

Prior to analysis, night-time sleep data were filtered removing observations within the timeframe of 8pm to 8am that were <3 hours in duration or >14 hours in duration. Night-time observations with sleep score of 0 and/or sleep efficiency score of 0 were removed, as were impossible values for covariates.

Dots in the diagram below indicate the start and end of individual sleep sessions detected by the watch. In the upper part of the figure (“Not combined”), each detected sleep interval is treated as an independent episode, and WASO is calculated solely from epochs classified by the device as wake within each discrete sleep session. In the lower part of the figure (“Combined sleep”), temporally adjacent sleep sessions within a night are merged into a single main sleep period after excluding outlier gaps between sessions, defined as gaps longer than 1.5 times the interquartile range of all between-session gaps. In this combined representation, WASO comprises both wake epochs occurring within sleep stages and the intervening awake time between merged sessions, illustrated by the red dotted line between duration A and duration B, so that total nightly WASO reflects all wakefulness from the first sleep onset after 20:00 until the final awakening based on the watch-derived classifications.

#### Age categories

Age, according to an individual’s user profile, was categorized as 20.0–24.9 years, 25.0–29.9 years, 30.0–34.9 years, 35.0–39.9 years, 40.0–44.9 years, 50.0–54.9 years, and 60.0–65.0 years.

**A)**

**Females**

**Males**

**B)**

Figure S1. Age distribution of (A) females and (B) males by country.

UK, United Kingdom, US, United States

Table S1. Age distribution of females overall and by country.

|  | **Total** | **France** | **Germany** | **South Korea** | **UK** | **US** |
| --- | --- | --- | --- | --- | --- | --- |
| **Age category, years** | **N = 192,500** | **N = 38,500** | **N = 38,500** | **N = 38,500** | **N = 38,500** | **N = 38,500** |
| 20–24 | 6607 (3.4) | 1281 (3.3) | 1357 (3.5) | 2211 (5.7) | 808 (2.1) | 950 (2.5) |
| 25–29 | 14,705 (7.6) | 3161 (8.2) | 2698 (7.0) | 3890 (10.1) | 2564 (6.7) | 2392 (6.2) |
| 30–34 | 22,019 (11.4) | 4128 (10.7) | 3639 (9.5) | 4442 (11.5) | 5175 (13.4) | 4635 (12.0) |
| 35–39 | 30,545 (15.9) | 6098 (15.8) | 6047 (15.7) | 4582 (11.9) | 6992 (18.2) | 6826 (17.7) |
| 40–44 | 31,742 (16.5) | 6525 (16.9) | 5675 (14.7) | 5828 (15.1) | 6620 (17.2) | 7094 (18.4) |
| 45–49 | 27,845 (14.5) | 5887 (15.3) | 5420 (14.1) | 5607 (14.6) | 5176 (13.4) | 5755 (14.9) |
| 50–54 | 24,435 (12.7) | 5164 (13.4) | 4834 (12.6) | 5251 (13.6) | 4720 (12.3) | 4466 (11.6) |
| 55–59 | 20,108 (10.4) | 3638 (9.4) | 4959 (12.9) | 3903 (10.1) | 3893 (10.1) | 3715 (9.6) |
| 60–65 | 14,494 (7.5) | 2618 (6.8) | 3871 (10.1) | 2786 (7.2) | 2,552 (6.6) | 2667 (6.9) |

**Data are n (%)**

UK, United Kingdom; US, United States

Table S2. Age distribution of males overall and by country.

|  | **Total** | **France** | **Germany** | **South Korea** | **UK** | **US** |
| --- | --- | --- | --- | --- | --- | --- |
| **Age category, years** | **N = 145,000** | **N = 29,000** | **N = 29,000** | **N = 29,000** | **N = 29,000** | **N = 29,000** |
| 20–24 | 7016 (4.8) | 1913 (6.6) | 1639 (5.7) | 1181 (4.1) | 1106 (3.8) | 1177 (4.1) |
| 25–29 | 13,378 (9.2) | 3380 (11.7) | 2595 (8.9) | 2256 (7.8) | 2563 (8.8) | 2584 (8.9) |
| 30–34 | 15,966 (11.0) | 3294 (11.4) | 2750 (9.5) | 2621 (9.0) | 3704 (12.8) | 3597 (12.4) |
| 35–39 | 17,116 (11.8) | 3327 (11.5) | 3308 (11.4) | 2538 (8.8) | 3945 (13.6) | 3998 (13.8) |
| 40–44 | 22,442 (15.5) | 4342 (15.0) | 3881 (13.4) | 4409 (15.2) | 4714 (16.3) | 5096 (17.6) |
| 45–49 | 20,784 (14.3) | 4002 (13.8) | 3822 (13.2) | 4690 (16.2) | 4074 (14.0) | 4196 (14.5) |
| 50–54 | 19,100 (13.2) | 3777 (13.0) | 3470 (12.0) | 4766 (16.4) | 3694 (12.7) | 3393 (11.7) |
| 55–59 | 16,604 (11.5) | 2910 (10.0) | 3974 (13.7) | 3723 (12.8) | 3129 (10.8) | 2868 (9.9) |
| 60–65 | 12,594 (8.7) | 2055 (7.1) | 3561 (12.3) | 2816 (9.7) | 2071 (7.1) | 2091 (7.2) |

**Data are n (%)**

UK, United Kingdom; US, United States

Table S3. BMI, and daily step count and physical activity time in women overall and by country.

|  |  | **Total** | **France** | **Germany** | **South Korea** | **UK** | **US** |
| --- | --- | --- | --- | --- | --- | --- | --- |
|  |  | **N = 192,500** | **N = 6607** | **N = 14,705** | **N = 22,019** | **N = 30,545** | **N = 31,742** |
| **BMI, kg/m^2^** | Mean (SD) | 27.6 (10.6) | 26.8 (9.9) | 27.8 (11.9) | 23.6 (5.1) | 29.3 (14.0) | 30.7 (8.0) |
|  | 18.5–24.9 (healthy weight), n (%) | 82,673 (42.9) | 17,773 (46.2) | 15,607 (40.5) | 27,612 (71.7) | 12,313 (32.0) | 9368 (24.3) |
|  | 25.0–29.9 (overweight), n (%) | 53,636 (27.9) | 11,509 (29.9) | 11,586 (30.1) | 8,046 (20.9) | 11,622 (30.2) | 10,873 (28.2) |
|  | ≥30 (obesity), n (%) | 56,191 (29.2) | 9,218 (23.9) | 11,307 (29.4) | 2,842 (7.4%) | 14,565 (37.8) | 18,259 (47.4) |
| **Daily step count** | Mean (SD) | 6,817.2 (3281.0) | 6588.7 (3030.8) | 7595.3 (3345.3) | 7454.5 (3416.8) | 6782.3 (3329.3) | 5614.3 (2862.6) |
|  | <5000 (sedentary), n (%) | 64,182 (33.3) | 13,257 (34.4) | 8,884 (23.1) | 9,513 (24.7) | 13,390 (34.8) | 19,138 (49.7) |
|  | 5000 to 7499 (low active), n (%) | 64,691 (33.6) | 13,978 (36.3) | 13,099 (34.0) | 13,205 (34.3) | 12,438 (32.3) | 11,971 (31.1) |
|  | 7500 to 9999 (somewhat active), n (%) | 37,930 (19.7) | 7166 (18.6) | 9363 (24.3) | ,072 (23.6) | 7371 (19.1) | 4958 (12.9) |
|  | >10,000 (active), n (%) | 16,180 (8.4) | 2707 (7.0) | 4413 (11.5) | 4115 (10.7) | 3314 (8.6) | 1631 (4.2) |
|  | >12,500 (highly active), n (%) | 9517 (4.9) | 1392 (3.6) | 2741 (7.1) | 2595 (6.7) | 1987 (5.2) | 802 (2.1) |
| **Physical activity time, minutes** | Mean (SD) | 75.7 (36.0) | 73.1 (32.8) | 85.3 (37.3) | 81.1 (36.3) | 74.8 (36.8) | 63.7 (32.4) |
|  | First tertile | 57.5 | 56.7 | 66.5 | 63.1 | 56.2 | 47 |
|  | Second tertile | 85.2 | 81.3 | 95.4 | 90.8 | 84.6 | 71.8 |
|  | Min | 0.3 | 0.6 | 0.8 | 1 | 1.2 | 0.3 |
|  | Max | 1182.2 | 619.2 | 1182.2 | 685.5 | 814.5 | 768.7 |
|  | Lower tertile (average active time ≤ first tertile) | 69,140 (35.9) | 14,392 (37.4) | 9,518 (24.7) | 10,999 (28.6) | 14,535 (37.8) | 19,696 (51.2) |
|  | Mid tertile (average active time >first tertile and ≤ second tertile) | 63,185 (32.8) | 13,530 (35.1) | 12,846 (33.4%) | 13,237 (34.4) | 12,188 (31.7) | 11,384 (29.6) |
|  | Upper tertile (average active time >second tertile) | 60,175 (31.3) | 10,578 (27.5) | 16,136 (41.9) | 14,264 (37.0) | 11,777 (30.6) | 7420 (19.3) |

**^a^** Tertile values were calculated using individual user's average active time.

BMI, body mass index; SD, standard deviation

Table S4. Mean and median values for TSPD, TST, and WASO in women overall and by 5-year age category.

|  | | **Age group, years** | | | | | | | | | |
| --- | --- | --- | --- | --- | --- | --- | --- | --- | --- | --- | --- |
|  | | **Total** | **20–24** | **25–29** | **30–34** | **35–39** | **40–44** | **45–49** | **50–54** | **55–59** | **60–65** |
|  | | **N=192,500** | **N=6607** | **N=14,705** | **N=22,019** | **N=30,545** | **N=31,742** | **N=27,845** | **N=24,435** | **N=20,108** | **N=14,494** |
| **TSPD** | Mean | 439.44 | 439.08 | 440.89 | 440.64 | 441.16 | 439.64 | 438.2 | 437.45 | 438.25 | 440.01 |
|  | SD | 92.03 | 101.05 | 97.51 | 94.09 | 91.08 | 90.77 | 90.74 | 91 | 90.38 | 90.63 |
|  | Q1 | 383 | 374 | 380 | 383 | 386 | 385 | 383 | 381 | 383 | 384 |
|  | Median | 442 | 441 | 443 | 444 | 445 | 443 | 440 | 439 | 440 | 443 |
|  | Q3 | 498 | 504 | 502 | 501 | 499 | 497 | 496 | 495 | 496 | 498 |
| **TST** | Mean | 391.19 | 396.79 | 397.15 | 394.24 | 394.35 | 393.62 | 391.5 | 387.07 | 384.48 | 383.52 |
|  | SD | 81.87 | 90.29 | 87.1 | 84.1 | 81.73 | 81.4 | 80.75 | 80.07 | 78.81 | 78.94 |
|  | Q1 | 341 | 339 | 343 | 342 | 345 | 344 | 342 | 338 | 336 | 335 |
|  | Median | 395 | 400 | 400 | 399 | 399 | 398 | 395 | 390 | 388 | 387 |
|  | Q3 | 444 | 456 | 453 | 449 | 448 | 447 | 444 | 439 | 436 | 435 |
| **WASO** | Mean | 48.25 | 42.29 | 43.74 | 46.39 | 46.81 | 46.02 | 46.7 | 50.38 | 53.77 | 56.49 |
|  | SD | 33.5 | 28.1 | 30.01 | 32.6 | 33.19 | 32.82 | 33.25 | 34.36 | 34.95 | 36.06 |
|  | Q1 | 25 | 24 | 24 | 25 | 24 | 24 | 24 | 27 | 30 | 31 |
|  | Median | 39 | 35 | 36 | 37 | 37 | 37 | 37 | 41 | 45 | 47 |
|  | Q3 | 61 | 52 | 54 | 58 | 59 | 58 | 59 | 64 | 68 | 71 |

Q1, first quartile; Q3, third quartile; SD, standard deviation; TSPD, total sleep period duration; TST, total sleep time; WASO, wakefulness after sleep onset

Table S5. Mean and median values for TSPD, TST, and WASO in men overall and by 5-year age category.

|  | | **Age group, years** | | | | | | | | | |
| --- | --- | --- | --- | --- | --- | --- | --- | --- | --- | --- | --- |
|  | | **Total** | **20–24** | **25–29** | **30–34** | **35–39** | **40–44** | **45–49** | **50–54** | **55–59** | **60–65** |
|  | | **N=145,000** | **N= 7016** | **N=13,378** | **N=15,966** | **N=17,116** | **N=22,442** | **N=20,784** | **N=19,100** | **N=16,604** | **N=12,594** |
| **TSPD** | Mean | 416.16 | 425.56 | 421.42 | 417.44 | 415.54 | 413.16 | 412.35 | 413.53 | 416.06 | 420.55 |
|  | SD | 90.85 | 94.84 | 92.78 | 91.4 | 90.64 | 90.42 | 89.83 | 90.31 | 90.03 | 89.94 |
|  | Q1 | 358 | 365 | 362 | 359 | 358 | 355 | 355 | 356 | 359 | 364 |
|  | Median | 418 | 427 | 423 | 420 | 418 | 415 | 414 | 414 | 417 | 422 |
|  | Q3 | 474 | 485 | 480 | 476 | 474 | 471 | 469 | 471 | 473 | 478 |
| **TST** | Mean | 370.09 | 383.93 | 379.31 | 374.24 | 371.4 | 369.15 | 367.35 | 365.82 | 365.62 | 365.74 |
|  | SD | 81.42 | 85.45 | 83.74 | 82.3 | 81.4 | 81.11 | 80.56 | 80.5 | 79.98 | 79.32 |
|  | Q1 | 318 | 330 | 326 | 322 | 319 | 317 | 316 | 314 | 315 | 315 |
|  | Median | 373 | 386 | 382 | 377 | 374 | 372 | 370 | 368 | 368 | 368 |
|  | Q3 | 423 | 439 | 433 | 428 | 425 | 422 | 420 | 418 | 418 | 418 |
| **WASO** | Mean | 46.07 | 41.63 | 42.11 | 43.2 | 44.14 | 44 | 45 | 47.71 | 50.44 | 54.8 |
|  | SD | 32.61 | 26.13 | 27.39 | 29.71 | 31.54 | 31.94 | 32.44 | 34.22 | 35.43 | 37.12 |
|  | Q1 | 24 | 25 | 24 | 24 | 23 | 23 | 23 | 24 | 26 | 28 |
|  | Median | 37 | 35 | 35 | 35 | 35 | 35 | 35 | 38 | 40 | 44 |
|  | Q3 | 57 | 51 | 51 | 53 | 55 | 55 | 56 | 60 | 65 | 71 |

Q1, first quartile; Q3, third quartile; SD, standard deviation; TSPD, total sleep period duration; TST, total sleep time; WASO, wakefulness after sleep onset

Table S6. Mean and median number of awakenings in women overall and by 5-year age category.

|  |  |  | **Age group, years** | | | | | | | | |
| --- | --- | --- | --- | --- | --- | --- | --- | --- | --- | --- | --- |
|  |  | **Total** | **20–24** | **25–29** | **30–34** | **35–39** | **40–44** | **45–49** | **50–54** | **55–59** | **60–65** |
|  |  | **N=145,000** | **N= 7016** | **N=13,378** | **N=15,966** | **N=17,116** | **N=22,442** | **N=20,784** | **N=19,100** | **N=16,604** | **N=12,594** |
| Number of awakenings | Mean | 16.71 | 18.22 | 17.82 | 17.21 | 16.64 | 16.49 | 16.29 | 16.38 | 16.52 | 16.5 |
|  | SD | 6.64 | 7.37 | 7.09 | 6.84 | 6.63 | 6.64 | 6.55 | 6.4 | 6.37 | 6.29 |
|  | Q1 | 12 | 13 | 13 | 12 | 12 | 12 | 12 | 12 | 12 | 12 |
|  | Median | 16 | 17 | 17 | 17 | 16 | 16 | 16 | 16 | 16 | 16 |
|  | Q3 | 20 | 22 | 22 | 21 | 20 | 20 | 20 | 20 | 20 | 20 |
| Number of awakenings (≥3 minutes) | Mean | 3.36 | 3.12 | 3.07 | 3.11 | 3.08 | 3.01 | 3.11 | 3.61 | 4.12 | 4.35 |
|  | SD | 2.42 | 2.36 | 2.3 | 2.32 | 2.29 | 2.27 | 2.29 | 2.47 | 2.57 | 2.6 |
|  | Q1 | 2 | 1 | 1 | 1 | 1 | 1 | 1 | 2 | 2 | 2 |
|  | Median | 3 | 3 | 3 | 3 | 3 | 3 | 3 | 3 | 4 | 4 |
|  | Q3 | 5 | 4 | 4 | 4 | 4 | 4 | 4 | 5 | 6 | 6 |

Q1, first quartile; Q3, third quartile; SD, standard deviation

Table S7. Estimate (95% CI) for difference in TSPD, TST, and WASO for age and other covariates compared with the respective reference group in females (regression model).

|  | **TSPD** | | | **TST** | | | **WASO** | | |
| --- | --- | --- | --- | --- | --- | --- | --- | --- | --- |
|  | **Estimate** | **Lower CI** | **Upper CI** | **Estimate** | **Lower CI** | **Upper CI** | **Estimate** | **Lower CI** | **Upper CI** |
| **Age, years** |  |  |  |  |  |  |  |  |  |
| 20–24 (reference) | – | – | – | – | – | – | – | – | – |
| 25–29 | −0.482 | −1.885 | 0.922 | −1.604 | −2.911 | −0.297 | 0.590542 | 0.212401 | 0.965923 |
| 30–34 | −2.246 | −3.579 | −0.914 | −5.608 | −6.848 | −4.367 | 1.895472 | 1.513924 | 2.257497 |
| 35–39 | −3.079 | −4.369 | −1.789 | −6.884 | −8.085 | −5.683 | 2.017449 | 1.661451 | 2.370321 |
| 40–44 | −3.599 | −4.884 | −2.315 | −6.681 | −7.877 | −5.486 | 1.367225 | 1.002202 | 1.735437 |
| 45–49 | −4.864 | −6.162 | −3.565 | −8.731 | −9.94 | −7.522 | 1.960414 | 1.587614 | 2.332675 |
| 50–54 | −5.39 | −6.705 | −4.075 | −13.039 | −14.263 | −11.815 | 5.439287 | 5.020593 | 5.835005 |
| 55–59 | −5.327 | −6.671 | −3.983 | −16.26 | −17.511 | −15.008 | 8.807055 | 8.37829 | 9.260532 |
| 60–65 | −3.556 | −4.96 | −2.151 | −17.294 | −18.602 | −15.986 | 11.24973 | 10.75537 | 11.73263 |
| **Country** |  |  |  |  |  |  |  |  |  |
| United States (reference) | – | – | – | – | – | – | – | – | – |
| Germany | 11.503 | 10.814 | 12.192 | 12.685 | 12.043 | 13.327 | 0.074577 | -0.10572 | 0.247926 |
| France | 19.436 | 18.744 | 20.129 | 19.719 | 19.074 | 20.364 | 0.768142 | 0.569248 | 0.965923 |
| United Kingdom | 16.023 | 15.339 | 16.706 | 15.309 | 14.672 | 15.946 | 1.478296 | 1.293737 | 1.661451 |
| South Korea | −27.103 | −27.83 | −26.376 | −24.66 | −25.337 | −23.984 | −1.02808 | −1.21393 | −0.83698 |
| **BMI** |  |  |  |  |  |  |  |  |  |
| Healthy weight (25.0–29.9 kg/m^2^)  (reference) | – | – | – | – | – | – | – | – | – |
| Overweight (25.0–29.9 kg/m^2^) | −9.388 | −9.94 | −8.836 | −10.886 | −11.4 | −10.372 | 0.2844 | 0.141459 | 0.426081 |
| Obesity (≥30 kg/m^2^) | −2.553 | −3.088 | −2.017 | −3.303 | −3.802 | −2.804 | 0.215471 | 0.070659 | 0.354712 |
| **Weekday/weekend** |  |  |  |  |  |  |  |  |  |
| Weekday (reference) | – | – | – | – | – | – | – | – | – |
| Weekend | 23.786 | 23.607 | 23.965 | 20.946 | 20.79 | 21.102 | 1.902037 | 1.846692 | 1.958282 |
| **Activity** |  |  |  |  |  |  |  |  |  |
| Lower tertile (reference) | – | – | – | – | – | – | – | – | – |
| Middle tertile | −0.865 | −1.261 | −0.469 | −0.153 | −0.498 | 0.192 | −0.38675 | −0.49068 | −0.28123 |
| Upper tertile | −2.114 | −2.622 | −1.607 | −1.051 | −1.493 | −0.609 | −0.56569 | −0.69887 | −0.45585 |
| **Step count** |  |  |  |  |  |  |  |  |  |
| Sedentary (reference) | – | – | – | – | – | – | – | – | – |
| Low | −2.527 | −2.985 | −2.069 | −.561 | −1.961 | −1.161 | −0.63724 | −0.73345 | −0.52546 |
| Somewhat | −1.48 | −1.882 | −1.078 | −0.879 | −1.229 | −0.529 | −0.37782 | −0.49068 | −0.28123 |
| Active | −4.981 | −5.548 | −4.415 | −3.462 | −3.956 | −2.968 | −0.90088 | −1.0431 | −0.76799 |
| High | −3.161 | −3.716 | −2.607 | −1.945 | −2.429 | −1.461 | −0.75939 | −0.90582 | −0.62961 |

BMI, body mass index; CI, confidence interval; SD, standard deviation; TSPD, total sleep period duration; TST, total sleep time; WASO, wakefulness after sleep onset

Table S8. Estimate (95% CI) for difference in TSPD, TST, and WASO for age and other covariates compared with the respective reference group in men (regression model).

|  | **TSPD** | | | **TST** | | | **WASO** | | |
| --- | --- | --- | --- | --- | --- | --- | --- | --- | --- |
|  | **Estimate** | **Lower CI** | **Upper CI** | **Estimate** | **Lower CI** | **Upper CI** | **Estimate** | **Lower CI** | **Upper CI** |
| **Age, years** |  |  |  |  |  |  |  |  |  |
| 20–24 (reference) | – | – | – | – | – | – | – | – | – |
| 25–29 | −2.74 | −4.134 | −1.347 | −3.358 | −4.684 | −2.032 | 0.163893 | −0.20824 | 0.526111 |
| 30–34 | −5.561 | −6.92 | −4.202 | −7.249 | −8.542 | −5.956 | 0.358699 | 0 | 0.738775 |
| 35–39 | −7.329 | −8.677 | −5.981 | −10.061 | −11.344 | −8.778 | 0.521096 | 0.174494 | 0.881261 |
| 40–44 | −7.812 | −9.114 | −6.51 | −10.608 | −11.847 | −9.369 | 0.299972 | −0.03479 | 0.632283 |
| 45–49 | −**7.878** | −9.192 | −6.564 | −11.703 | −12.954 | −10.452 | 1.044349 | 0.703242 | 1.42069 |
| 50–54 | −6.119 | −7.448 | −4.79 | −12.777 | −14.042 | −11.512 | 3.178512 | 2.786396 | 3.545927 |
| 55–59 | −4.542 | −5.896 | −3.188 | −14.053 | −15.341 | −12.764 | 5.573588 | 5.151272 | 5.999368 |
| 60–65 | −0.196 | −1.609 | 1.216 | −14.16 | −15.505 | −12.816 | 9.43876 | 8.9585 | 9.932114 |
| **Country** |  |  |  |  |  |  |  |  |  |
| United States (reference) | – | – | – | – | – | – | – | – | – |
| Germany | 10.858 | 10.071 | 11.646 | 12.18 | 11.431 | 12.93 | −0.32573 | −0.51828 | −0.10428 |
| France | 13.72 | 12.926 | 14.513 | 13.535 | 12.78 | 14.29 | 0.630369 | 0.420256 | 0.845586 |
| United Kingdom | 13.888 | 13.103 | 14.673 | 12.513 | 11.766 | 13.26 | 1.747753 | 1.52955 | 1.968272 |
| South Korea | −24.849 | −25.664 | −24.034 | −24.652 | −25.427 | −23.876 | 0.796386 | 0.561466 | 1.024319 |
| **BMI** |  |  |  |  |  |  |  |  |  |
| Healthy weight (25.0–29.9 kg/m^2^)  (reference) | – | – | – | – | – | – | – | – | – |
| Overweight (25.0–29.9 kg/m^2^) | −14.308 | −14.983 | −13.633 | −14.077 | −14.719 | −13.434 | −1.02479 | −1.19733 | −0.8595 |
| Obesity (≥30 kg/m^2^) | −6.574 | −7.167 | −5.981 | −5.478 | −6.043 | −4.914 | −1.12323 | −1.26449 | −0.96121 |
| **Weekday/weekend** |  |  |  |  |  |  |  |  |  |
| Weekday (reference) | – | – | – | – | – | – | – | – | – |
| Weekend | 24.738 | 24.533 | 24.944 | 21.621 | 21.442 | 21.621 | 2.209495 | 2.152632 | 2.209495 |
| **Activity** |  |  |  |  |  |  |  |  |  |
| Lower tertile (reference) | – | – | – | – | – | – | – | – | – |
| Middle tertile | −0.556 | −0.992 | −0.12 | −0.402 | −0.782 | −0.402 | −0.03272 | −0.13897 | 0.069693 |
| Upper tertile | −1.597 | −2.19 | −1.003 | −1.301 | −1.819 | −1.301 | −0.02329 | −0.17362 | 0.139525 |
| **Step count** |  |  |  |  |  |  |  |  |  |
| Sedentary (reference) | – | – | – | – | – | – | – | – | – |
| Low | −0.607 | −1.033 | −0.18 | −0.224 | −0.595 | 0.148 | −0.15986 | −0.27738 | −0.06955 |
| Somewhat active | −1.467 | −1.979 | −0.955 | −0.899 | −1.346 | −0.453 | −0.23735 | −0.38083 | −0.23735 |
| Active | −3.709 | −4.373 | −3.709 | −2.705 | −3.285 | −2.124 | −0.45715 | −0.621 | −0.27738 |
| High | −1.842 | −2.485 | −1.199 | −1.214 | −1.775 | −0.652 | −0.28197 | −0.44962 | −0.10428 |

BMI, body mass index; CI, confidence interval; SD, standard deviation; TSPD, total sleep period duration; TST, total sleep time; WASO, wakefulness after sleep onset
