# Supplementary material for: Sleep pattern differences in women at midlife: a large-scale multi-country smartwatch study (SONAR)
Source: Sleep. 2026 Jul 28;49(10):zsag197. doi: 10.1093/sleep/zsag197 (PMC13658266; doi:10.1093/sleep/zsag197)
Supplement: Supplementary file 1 [file supplementary_material_zsag197.zip › Supplementary Figure S1.docx]

**A)**

**Females**

**Males**

**B)**

**Figure S1.** Age distribution of **(A)** females and **(B)** males by country.

UK, United Kingdom, US, United States
