# Supplementary material for: Sleep pattern differences in women at midlife: a large-scale multi-country smartwatch study (SONAR)
Source: Sleep. 2026 Jul 28;49(10):zsag197. doi: 10.1093/sleep/zsag197 (PMC13658266; doi:10.1093/sleep/zsag197)
Supplement: Supplementary file 1 [file supplementary_material_zsag197.zip › Supplementary Methods.docx]

## Supplementary Methods

**Preparation of sleep data prior to analysis**

Prior to analysis, night-time sleep data were filtered removing observations within the timeframe of 8pm to 8am that were <3 hours in duration or >14 hours in duration. Night-time observations with sleep score of 0 and/or sleep efficiency score of 0 were removed, as were impossible values for covariates.

Dots in the diagram below indicate the start and end of individual sleep sessions detected by the watch. In the upper part of the figure (“Not combined”), each detected sleep interval is treated as an independent episode, and WASO is calculated solely from epochs classified by the device as wake within each discrete sleep session. In the lower part of the figure (“Combined sleep”), temporally adjacent sleep sessions within a night are merged into a single main sleep period after excluding outlier gaps between sessions, defined as gaps longer than 1.5 times the interquartile range of all between-session gaps. In this combined representation, WASO comprises both wake epochs occurring within sleep stages and the intervening awake time between merged sessions, illustrated by the red dotted line between duration A and duration B, so that total nightly WASO reflects all wakefulness from the first sleep onset after 20:00 until the final awakening based on the watch-derived classifications.

**Age categories**

Age, according to an individual’s user profile, was categorized as 20.0–24.9 years, 25.0–29.9 years, 30.0–34.9 years, 35.0–39.9 years, 40.0–44.9 years, 50.0–54.9 years, and 60.0–65.0 years.
