# Supplementary material for: Sleep pattern differences in women at midlife: a large-scale multi-country smartwatch study (SONAR)
Source: Sleep. 2026 Jul 28;49(10):zsag197. doi: 10.1093/sleep/zsag197 (PMC13658266; doi:10.1093/sleep/zsag197)
Supplement: Supplementary file 1 [file supplementary_material_zsag197.zip › Supplementary Table S1.docx]

**Table S1.** Age distribution of females overall and by country.

|  | **Total** | **France** | **Germany** | **South Korea** | **UK** | **US** |
| --- | --- | --- | --- | --- | --- | --- |
| **Age category, years** | **N = 192,500** | **N = 38,500** | **N = 38,500** | **N = 38,500** | **N = 38,500** | **N = 38,500** |
| 20–24 | 6607 (3.4) | 1281 (3.3) | 1357 (3.5) | 2211 (5.7) | 808 (2.1) | 950 (2.5) |
| 25–29 | 14,705 (7.6) | 3161 (8.2) | 2698 (7.0) | 3890 (10.1) | 2564 (6.7) | 2392 (6.2) |
| 30–34 | 22,019 (11.4) | 4128 (10.7) | 3639 (9.5) | 4442 (11.5) | 5175 (13.4) | 4635 (12.0) |
| 35–39 | 30,545 (15.9) | 6098 (15.8) | 6047 (15.7) | 4582 (11.9) | 6992 (18.2) | 6826 (17.7) |
| 40–44 | 31,742 (16.5) | 6525 (16.9) | 5675 (14.7) | 5828 (15.1) | 6620 (17.2) | 7094 (18.4) |
| 45–49 | 27,845 (14.5) | 5887 (15.3) | 5420 (14.1) | 5607 (14.6) | 5176 (13.4) | 5755 (14.9) |
| 50–54 | 24,435 (12.7) | 5164 (13.4) | 4834 (12.6) | 5251 (13.6) | 4720 (12.3) | 4466 (11.6) |
| 55–59 | 20,108 (10.4) | 3638 (9.4) | 4959 (12.9) | 3903 (10.1) | 3893 (10.1) | 3715 (9.6) |
| 60–65 | 14,494 (7.5) | 2618 (6.8) | 3871 (10.1) | 2786 (7.2) | 2,552 (6.6) | 2667 (6.9) |

**Data are n (%)**

UK, United Kingdom; US, United States
