# Supplementary material for: Sleep pattern differences in women at midlife: a large-scale multi-country smartwatch study (SONAR)
Source: Sleep. 2026 Jul 28;49(10):zsag197. doi: 10.1093/sleep/zsag197 (PMC13658266; doi:10.1093/sleep/zsag197)
Supplement: Supplementary file 1 [file supplementary_material_zsag197.zip › Supplementary Table S2.docx]

**Table S2.** Age distribution of males overall and by country.

|  | **Total** | **France** | **Germany** | **South Korea** | **UK** | **US** |
| --- | --- | --- | --- | --- | --- | --- |
| **Age category, years** | **N = 145,000** | **N = 29,000** | **N = 29,000** | **N = 29,000** | **N = 29,000** | **N = 29,000** |
| 20–24 | 7016 (4.8) | 1913 (6.6) | 1639 (5.7) | 1181 (4.1) | 1106 (3.8) | 1177 (4.1) |
| 25–29 | 13,378 (9.2) | 3380 (11.7) | 2595 (8.9) | 2256 (7.8) | 2563 (8.8) | 2584 (8.9) |
| 30–34 | 15,966 (11.0) | 3294 (11.4) | 2750 (9.5) | 2621 (9.0) | 3704 (12.8) | 3597 (12.4) |
| 35–39 | 17,116 (11.8) | 3327 (11.5) | 3308 (11.4) | 2538 (8.8) | 3945 (13.6) | 3998 (13.8) |
| 40–44 | 22,442 (15.5) | 4342 (15.0) | 3881 (13.4) | 4409 (15.2) | 4714 (16.3) | 5096 (17.6) |
| 45–49 | 20,784 (14.3) | 4002 (13.8) | 3822 (13.2) | 4690 (16.2) | 4074 (14.0) | 4196 (14.5) |
| 50–54 | 19,100 (13.2) | 3777 (13.0) | 3470 (12.0) | 4766 (16.4) | 3694 (12.7) | 3393 (11.7) |
| 55–59 | 16,604 (11.5) | 2910 (10.0) | 3974 (13.7) | 3723 (12.8) | 3129 (10.8) | 2868 (9.9) |
| 60–65 | 12,594 (8.7) | 2055 (7.1) | 3561 (12.3) | 2816 (9.7) | 2071 (7.1) | 2091 (7.2) |

**Data are n (%)**

UK, United Kingdom; US, United States
