# Supplementary material for: Sleep pattern differences in women at midlife: a large-scale multi-country smartwatch study (SONAR)
Source: Sleep. 2026 Jul 28;49(10):zsag197. doi: 10.1093/sleep/zsag197 (PMC13658266; doi:10.1093/sleep/zsag197)
Supplement: Supplementary file 1 [file supplementary_material_zsag197.zip › Supplementary Table S3.docx]

**Table S3.** BMI, and daily step count and physical activity time in women overall and by country.

|  |  | **Total** | **France** | **Germany** | **South Korea** | **UK** | **US** |
| --- | --- | --- | --- | --- | --- | --- | --- |
|  |  | **N = 192,500** | **N = 6607** | **N = 14,705** | **N = 22,019** | **N = 30,545** | **N = 31,742** |
| **BMI, kg/m^2^** | Mean (SD) | 27.6 (10.6) | 26.8 (9.9) | 27.8 (11.9) | 23.6 (5.1) | 29.3 (14.0) | 30.7 (8.0) |
|  | 18.5–24.9 (healthy weight), n (%) | 82,673 (42.9) | 17,773 (46.2) | 15,607 (40.5) | 27,612 (71.7) | 12,313 (32.0) | 9368 (24.3) |
|  | 25.0–29.9 (overweight), n (%) | 53,636 (27.9) | 11,509 (29.9) | 11,586 (30.1) | 8,046 (20.9) | 11,622 (30.2) | 10,873 (28.2) |
|  | ≥30 (obesity), n (%) | 56,191 (29.2) | 9,218 (23.9) | 11,307 (29.4) | 2,842 (7.4%) | 14,565 (37.8) | 18,259 (47.4) |
| **Daily step count** | Mean (SD) | 6,817.2 (3281.0) | 6588.7 (3030.8) | 7595.3 (3345.3) | 7454.5 (3416.8) | 6782.3 (3329.3) | 5614.3 (2862.6) |
|  | <5000 (sedentary), n (%) | 64,182 (33.3) | 13,257 (34.4) | 8,884 (23.1) | 9,513 (24.7) | 13,390 (34.8) | 19,138 (49.7) |
|  | 5000 to 7499 (low active), n (%) | 64,691 (33.6) | 13,978 (36.3) | 13,099 (34.0) | 13,205 (34.3) | 12,438 (32.3) | 11,971 (31.1) |
|  | 7500 to 9999 (somewhat active), n (%) | 37,930 (19.7) | 7166 (18.6) | 9363 (24.3) | ,072 (23.6) | 7371 (19.1) | 4958 (12.9) |
|  | >10,000 (active), n (%) | 16,180 (8.4) | 2707 (7.0) | 4413 (11.5) | 4115 (10.7) | 3314 (8.6) | 1631 (4.2) |
|  | >12,500 (highly active), n (%) | 9517 (4.9) | 1392 (3.6) | 2741 (7.1) | 2595 (6.7) | 1987 (5.2) | 802 (2.1) |
| **Physical activity time, minutes** | Mean (SD) | 75.7 (36.0) | 73.1 (32.8) | 85.3 (37.3) | 81.1 (36.3) | 74.8 (36.8) | 63.7 (32.4) |
|  | First tertile | 57.5 | 56.7 | 66.5 | 63.1 | 56.2 | 47 |
|  | Second tertile | 85.2 | 81.3 | 95.4 | 90.8 | 84.6 | 71.8 |
|  | Min | 0.3 | 0.6 | 0.8 | 1 | 1.2 | 0.3 |
|  | Max | 1182.2 | 619.2 | 1182.2 | 685.5 | 814.5 | 768.7 |
|  | Lower tertile (average active time ≤ first tertile) | 69,140 (35.9) | 14,392 (37.4) | 9,518 (24.7) | 10,999 (28.6) | 14,535 (37.8) | 19,696 (51.2) |
|  | Mid tertile (average active time >first tertile and ≤ second tertile) | 63,185 (32.8) | 13,530 (35.1) | 12,846 (33.4%) | 13,237 (34.4) | 12,188 (31.7) | 11,384 (29.6) |
|  | Upper tertile (average active time >second tertile) | 60,175 (31.3) | 10,578 (27.5) | 16,136 (41.9) | 14,264 (37.0) | 11,777 (30.6) | 7420 (19.3) |

**^a^** Tertile values were calculated using individual user's average active time.

BMI, body mass index; SD, standard deviation
