# Supplementary material for: Sleep pattern differences in women at midlife: a large-scale multi-country smartwatch study (SONAR)
Source: Sleep. 2026 Jul 28;49(10):zsag197. doi: 10.1093/sleep/zsag197 (PMC13658266; doi:10.1093/sleep/zsag197)
Supplement: Supplementary file 1 [file supplementary_material_zsag197.zip › Supplementary Table S4.docx]

**Table S4.** Mean and median values for TSPD, TST, and WASO in women overall and by 5-year age category.

|  | | **Age group, years** | | | | | | | | | |
| --- | --- | --- | --- | --- | --- | --- | --- | --- | --- | --- | --- |
|  | | **Total** | **20–24** | **25–29** | **30–34** | **35–39** | **40–44** | **45–49** | **50–54** | **55–59** | **60–65** |
|  | | **N=192,500** | **N=6607** | **N=14,705** | **N=22,019** | **N=30,545** | **N=31,742** | **N=27,845** | **N=24,435** | **N=20,108** | **N=14,494** |
| **TSPD** | Mean | 439.44 | 439.08 | 440.89 | 440.64 | 441.16 | 439.64 | 438.2 | 437.45 | 438.25 | 440.01 |
|  | SD | 92.03 | 101.05 | 97.51 | 94.09 | 91.08 | 90.77 | 90.74 | 91 | 90.38 | 90.63 |
|  | Q1 | 383 | 374 | 380 | 383 | 386 | 385 | 383 | 381 | 383 | 384 |
|  | Median | 442 | 441 | 443 | 444 | 445 | 443 | 440 | 439 | 440 | 443 |
|  | Q3 | 498 | 504 | 502 | 501 | 499 | 497 | 496 | 495 | 496 | 498 |
| **TST** | Mean | 391.19 | 396.79 | 397.15 | 394.24 | 394.35 | 393.62 | 391.5 | 387.07 | 384.48 | 383.52 |
|  | SD | 81.87 | 90.29 | 87.1 | 84.1 | 81.73 | 81.4 | 80.75 | 80.07 | 78.81 | 78.94 |
|  | Q1 | 341 | 339 | 343 | 342 | 345 | 344 | 342 | 338 | 336 | 335 |
|  | Median | 395 | 400 | 400 | 399 | 399 | 398 | 395 | 390 | 388 | 387 |
|  | Q3 | 444 | 456 | 453 | 449 | 448 | 447 | 444 | 439 | 436 | 435 |
| **WASO** | Mean | 48.25 | 42.29 | 43.74 | 46.39 | 46.81 | 46.02 | 46.7 | 50.38 | 53.77 | 56.49 |
|  | SD | 33.5 | 28.1 | 30.01 | 32.6 | 33.19 | 32.82 | 33.25 | 34.36 | 34.95 | 36.06 |
|  | Q1 | 25 | 24 | 24 | 25 | 24 | 24 | 24 | 27 | 30 | 31 |
|  | Median | 39 | 35 | 36 | 37 | 37 | 37 | 37 | 41 | 45 | 47 |
|  | Q3 | 61 | 52 | 54 | 58 | 59 | 58 | 59 | 64 | 68 | 71 |

Q1, first quartile; Q3, third quartile; SD, standard deviation; TSPD, total sleep period duration; TST, total sleep time; WASO, wakefulness after sleep onset
