# Supplementary material for: Sleep pattern differences in women at midlife: a large-scale multi-country smartwatch study (SONAR)
Source: Sleep. 2026 Jul 28;49(10):zsag197. doi: 10.1093/sleep/zsag197 (PMC13658266; doi:10.1093/sleep/zsag197)
Supplement: Supplementary file 1 [file supplementary_material_zsag197.zip › Supplementary Table S5.docx]

**Table S5.** Mean and median values for TSPD, TST, and WASO in men overall and by 5-year age category.

|  | | **Age group, years** | | | | | | | | | |
| --- | --- | --- | --- | --- | --- | --- | --- | --- | --- | --- | --- |
|  | | **Total** | **20–24** | **25–29** | **30–34** | **35–39** | **40–44** | **45–49** | **50–54** | **55–59** | **60–65** |
|  | | **N=145,000** | **N= 7016** | **N=13,378** | **N=15,966** | **N=17,116** | **N=22,442** | **N=20,784** | **N=19,100** | **N=16,604** | **N=12,594** |
| **TSPD** | Mean | 416.16 | 425.56 | 421.42 | 417.44 | 415.54 | 413.16 | 412.35 | 413.53 | 416.06 | 420.55 |
|  | SD | 90.85 | 94.84 | 92.78 | 91.4 | 90.64 | 90.42 | 89.83 | 90.31 | 90.03 | 89.94 |
|  | Q1 | 358 | 365 | 362 | 359 | 358 | 355 | 355 | 356 | 359 | 364 |
|  | Median | 418 | 427 | 423 | 420 | 418 | 415 | 414 | 414 | 417 | 422 |
|  | Q3 | 474 | 485 | 480 | 476 | 474 | 471 | 469 | 471 | 473 | 478 |
| **TST** | Mean | 370.09 | 383.93 | 379.31 | 374.24 | 371.4 | 369.15 | 367.35 | 365.82 | 365.62 | 365.74 |
|  | SD | 81.42 | 85.45 | 83.74 | 82.3 | 81.4 | 81.11 | 80.56 | 80.5 | 79.98 | 79.32 |
|  | Q1 | 318 | 330 | 326 | 322 | 319 | 317 | 316 | 314 | 315 | 315 |
|  | Median | 373 | 386 | 382 | 377 | 374 | 372 | 370 | 368 | 368 | 368 |
|  | Q3 | 423 | 439 | 433 | 428 | 425 | 422 | 420 | 418 | 418 | 418 |
| **WASO** | Mean | 46.07 | 41.63 | 42.11 | 43.2 | 44.14 | 44 | 45 | 47.71 | 50.44 | 54.8 |
|  | SD | 32.61 | 26.13 | 27.39 | 29.71 | 31.54 | 31.94 | 32.44 | 34.22 | 35.43 | 37.12 |
|  | Q1 | 24 | 25 | 24 | 24 | 23 | 23 | 23 | 24 | 26 | 28 |
|  | Median | 37 | 35 | 35 | 35 | 35 | 35 | 35 | 38 | 40 | 44 |
|  | Q3 | 57 | 51 | 51 | 53 | 55 | 55 | 56 | 60 | 65 | 71 |

Q1, first quartile; Q3, third quartile; SD, standard deviation; TSPD, total sleep period duration; TST, total sleep time; WASO, wakefulness after sleep onset
