# Supplementary material for: Sleep pattern differences in women at midlife: a large-scale multi-country smartwatch study (SONAR)
Source: Sleep. 2026 Jul 28;49(10):zsag197. doi: 10.1093/sleep/zsag197 (PMC13658266; doi:10.1093/sleep/zsag197)
Supplement: Supplementary file 1 [file supplementary_material_zsag197.zip › Supplementary Table S6.docx]

**Table S6.** Mean and median number of awakenings in women overall and by 5-year age category.

|  |  |  | **Age group, years** | | | | | | | | |
| --- | --- | --- | --- | --- | --- | --- | --- | --- | --- | --- | --- |
|  |  | **Total** | **20–24** | **25–29** | **30–34** | **35–39** | **40–44** | **45–49** | **50–54** | **55–59** | **60–65** |
|  |  | **N=145,000** | **N= 7016** | **N=13,378** | **N=15,966** | **N=17,116** | **N=22,442** | **N=20,784** | **N=19,100** | **N=16,604** | **N=12,594** |
| Number of awakenings | Mean | 16.71 | 18.22 | 17.82 | 17.21 | 16.64 | 16.49 | 16.29 | 16.38 | 16.52 | 16.5 |
|  | SD | 6.64 | 7.37 | 7.09 | 6.84 | 6.63 | 6.64 | 6.55 | 6.4 | 6.37 | 6.29 |
|  | Q1 | 12 | 13 | 13 | 12 | 12 | 12 | 12 | 12 | 12 | 12 |
|  | Median | 16 | 17 | 17 | 17 | 16 | 16 | 16 | 16 | 16 | 16 |
|  | Q3 | 20 | 22 | 22 | 21 | 20 | 20 | 20 | 20 | 20 | 20 |
| Number of awakenings (≥3 minutes) | Mean | 3.36 | 3.12 | 3.07 | 3.11 | 3.08 | 3.01 | 3.11 | 3.61 | 4.12 | 4.35 |
|  | SD | 2.42 | 2.36 | 2.3 | 2.32 | 2.29 | 2.27 | 2.29 | 2.47 | 2.57 | 2.6 |
|  | Q1 | 2 | 1 | 1 | 1 | 1 | 1 | 1 | 2 | 2 | 2 |
|  | Median | 3 | 3 | 3 | 3 | 3 | 3 | 3 | 3 | 4 | 4 |
|  | Q3 | 5 | 4 | 4 | 4 | 4 | 4 | 4 | 5 | 6 | 6 |

Q1, first quartile; Q3, third quartile; SD, standard deviation
