# Supplementary material for: Sleep pattern differences in women at midlife: a large-scale multi-country smartwatch study (SONAR)
Source: Sleep. 2026 Jul 28;49(10):zsag197. doi: 10.1093/sleep/zsag197 (PMC13658266; doi:10.1093/sleep/zsag197)
Supplement: Supplementary file 1 [file supplementary_material_zsag197.zip › Supplementary Table S7.docx]

**Table S7.** Estimate (95% CI) for difference in TSPD, TST, and WASO for age and other covariates compared with the respective reference group in females (regression model).

|  | **TSPD** | | | **TST** | | | **WASO** | | |
| --- | --- | --- | --- | --- | --- | --- | --- | --- | --- |
|  | **Estimate** | **Lower CI** | **Upper CI** | **Estimate** | **Lower CI** | **Upper CI** | **Estimate** | **Lower CI** | **Upper CI** |
| **Age, years** |  |  |  |  |  |  |  |  |  |
| 20–24 (reference) | – | – | – | – | – | – | – | – | – |
| 25–29 | −0.482 | −1.885 | 0.922 | −1.604 | −2.911 | −0.297 | 0.590542 | 0.212401 | 0.965923 |
| 30–34 | −2.246 | −3.579 | −0.914 | −5.608 | −6.848 | −4.367 | 1.895472 | 1.513924 | 2.257497 |
| 35–39 | −3.079 | −4.369 | −1.789 | −6.884 | −8.085 | −5.683 | 2.017449 | 1.661451 | 2.370321 |
| 40–44 | −3.599 | −4.884 | −2.315 | −6.681 | −7.877 | −5.486 | 1.367225 | 1.002202 | 1.735437 |
| 45–49 | −4.864 | −6.162 | −3.565 | −8.731 | −9.94 | −7.522 | 1.960414 | 1.587614 | 2.332675 |
| 50–54 | −5.39 | −6.705 | −4.075 | −13.039 | −14.263 | −11.815 | 5.439287 | 5.020593 | 5.835005 |
| 55–59 | −5.327 | −6.671 | −3.983 | −16.26 | −17.511 | −15.008 | 8.807055 | 8.37829 | 9.260532 |
| 60–65 | −3.556 | −4.96 | −2.151 | −17.294 | −18.602 | −15.986 | 11.24973 | 10.75537 | 11.73263 |
| **Country** |  |  |  |  |  |  |  |  |  |
| United States (reference) | – | – | – | – | – | – | – | – | – |
| Germany | 11.503 | 10.814 | 12.192 | 12.685 | 12.043 | 13.327 | 0.074577 | -0.10572 | 0.247926 |
| France | 19.436 | 18.744 | 20.129 | 19.719 | 19.074 | 20.364 | 0.768142 | 0.569248 | 0.965923 |
| United Kingdom | 16.023 | 15.339 | 16.706 | 15.309 | 14.672 | 15.946 | 1.478296 | 1.293737 | 1.661451 |
| South Korea | −27.103 | −27.83 | −26.376 | −24.66 | −25.337 | −23.984 | −1.02808 | −1.21393 | −0.83698 |
| **BMI** |  |  |  |  |  |  |  |  |  |
| Healthy weight (25.0–29.9 kg/m^2^)  (reference) | – | – | – | – | – | – | – | – | – |
| Overweight (25.0–29.9 kg/m^2^) | −9.388 | −9.94 | −8.836 | −10.886 | −11.4 | −10.372 | 0.2844 | 0.141459 | 0.426081 |
| Obesity (≥30 kg/m^2^) | −2.553 | −3.088 | −2.017 | −3.303 | −3.802 | −2.804 | 0.215471 | 0.070659 | 0.354712 |
| **Weekday/weekend** |  |  |  |  |  |  |  |  |  |
| Weekday (reference) | – | – | – | – | – | – | – | – | – |
| Weekend | 23.786 | 23.607 | 23.965 | 20.946 | 20.79 | 21.102 | 1.902037 | 1.846692 | 1.958282 |
| **Activity** |  |  |  |  |  |  |  |  |  |
| Lower tertile (reference) | – | – | – | – | – | – | – | – | – |
| Middle tertile | −0.865 | −1.261 | −0.469 | −0.153 | −0.498 | 0.192 | −0.38675 | −0.49068 | −0.28123 |
| Upper tertile | −2.114 | −2.622 | −1.607 | −1.051 | −1.493 | −0.609 | −0.56569 | −0.69887 | −0.45585 |
| **Step count** |  |  |  |  |  |  |  |  |  |
| Sedentary (reference) | – | – | – | – | – | – | – | – | – |
| Low | −2.527 | −2.985 | −2.069 | −.561 | −1.961 | −1.161 | −0.63724 | −0.73345 | −0.52546 |
| Somewhat | −1.48 | −1.882 | −1.078 | −0.879 | −1.229 | −0.529 | −0.37782 | −0.49068 | −0.28123 |
| Active | −4.981 | −5.548 | −4.415 | −3.462 | −3.956 | −2.968 | −0.90088 | −1.0431 | −0.76799 |
| High | −3.161 | −3.716 | −2.607 | −1.945 | −2.429 | −1.461 | −0.75939 | −0.90582 | −0.62961 |

BMI, body mass index; CI, confidence interval; SD, standard deviation; TSPD, total sleep period duration; TST, total sleep time; WASO, wakefulness after sleep onset
