# Supplementary material for: Sleep pattern differences in women at midlife: a large-scale multi-country smartwatch study (SONAR)
Source: Sleep. 2026 Jul 28;49(10):zsag197. doi: 10.1093/sleep/zsag197 (PMC13658266; doi:10.1093/sleep/zsag197)
Supplement: Supplementary file 1 [file supplementary_material_zsag197.zip › Supplementary Table S8.docx]

**Table S8.** Estimate (95% CI) for difference in TSPD, TST, and WASO for age and other covariates compared with the respective reference group in men (regression model).

|  | **TSPD** | | | **TST** | | | **WASO** | | |
| --- | --- | --- | --- | --- | --- | --- | --- | --- | --- |
|  | **Estimate** | **Lower CI** | **Upper CI** | **Estimate** | **Lower CI** | **Upper CI** | **Estimate** | **Lower CI** | **Upper CI** |
| **Age, years** |  |  |  |  |  |  |  |  |  |
| 20–24 (reference) | – | – | – | – | – | – | – | – | – |
| 25–29 | −2.74 | −4.134 | −1.347 | −3.358 | −4.684 | −2.032 | 0.163893 | −0.20824 | 0.526111 |
| 30–34 | −5.561 | −6.92 | −4.202 | −7.249 | −8.542 | −5.956 | 0.358699 | 0 | 0.738775 |
| 35–39 | −7.329 | −8.677 | −5.981 | −10.061 | −11.344 | −8.778 | 0.521096 | 0.174494 | 0.881261 |
| 40–44 | −7.812 | −9.114 | −6.51 | −10.608 | −11.847 | −9.369 | 0.299972 | −0.03479 | 0.632283 |
| 45–49 | −**7.878** | −9.192 | −6.564 | −11.703 | −12.954 | −10.452 | 1.044349 | 0.703242 | 1.42069 |
| 50–54 | −6.119 | −7.448 | −4.79 | −12.777 | −14.042 | −11.512 | 3.178512 | 2.786396 | 3.545927 |
| 55–59 | −4.542 | −5.896 | −3.188 | −14.053 | −15.341 | −12.764 | 5.573588 | 5.151272 | 5.999368 |
| 60–65 | −0.196 | −1.609 | 1.216 | −14.16 | −15.505 | −12.816 | 9.43876 | 8.9585 | 9.932114 |
| **Country** |  |  |  |  |  |  |  |  |  |
| United States (reference) | – | – | – | – | – | – | – | – | – |
| Germany | 10.858 | 10.071 | 11.646 | 12.18 | 11.431 | 12.93 | −0.32573 | −0.51828 | −0.10428 |
| France | 13.72 | 12.926 | 14.513 | 13.535 | 12.78 | 14.29 | 0.630369 | 0.420256 | 0.845586 |
| United Kingdom | 13.888 | 13.103 | 14.673 | 12.513 | 11.766 | 13.26 | 1.747753 | 1.52955 | 1.968272 |
| South Korea | −24.849 | −25.664 | −24.034 | −24.652 | −25.427 | −23.876 | 0.796386 | 0.561466 | 1.024319 |
| **BMI** |  |  |  |  |  |  |  |  |  |
| Healthy weight (25.0–29.9 kg/m^2^)  (reference) | – | – | – | – | – | – | – | – | – |
| Overweight (25.0–29.9 kg/m^2^) | −14.308 | −14.983 | −13.633 | −14.077 | −14.719 | −13.434 | −1.02479 | −1.19733 | −0.8595 |
| Obesity (≥30 kg/m^2^) | −6.574 | −7.167 | −5.981 | −5.478 | −6.043 | −4.914 | −1.12323 | −1.26449 | −0.96121 |
| **Weekday/weekend** |  |  |  |  |  |  |  |  |  |
| Weekday (reference) | – | – | – | – | – | – | – | – | – |
| Weekend | 24.738 | 24.533 | 24.944 | 21.621 | 21.442 | 21.621 | 2.209495 | 2.152632 | 2.209495 |
| **Activity** |  |  |  |  |  |  |  |  |  |
| Lower tertile (reference) | – | – | – | – | – | – | – | – | – |
| Middle tertile | −0.556 | −0.992 | −0.12 | −0.402 | −0.782 | −0.402 | −0.03272 | −0.13897 | 0.069693 |
| Upper tertile | −1.597 | −2.19 | −1.003 | −1.301 | −1.819 | −1.301 | −0.02329 | −0.17362 | 0.139525 |
| **Step count** |  |  |  |  |  |  |  |  |  |
| Sedentary (reference) | – | – | – | – | – | – | – | – | – |
| Low | −0.607 | −1.033 | −0.18 | −0.224 | −0.595 | 0.148 | −0.15986 | −0.27738 | −0.06955 |
| Somewhat active | −1.467 | −1.979 | −0.955 | −0.899 | −1.346 | −0.453 | −0.23735 | −0.38083 | −0.23735 |
| Active | −3.709 | −4.373 | −3.709 | −2.705 | −3.285 | −2.124 | −0.45715 | −0.621 | −0.27738 |
| High | −1.842 | −2.485 | −1.199 | −1.214 | −1.775 | −0.652 | −0.28197 | −0.44962 | −0.10428 |

BMI, body mass index; CI, confidence interval; SD, standard deviation; TSPD, total sleep period duration; TST, total sleep time; WASO, wakefulness after sleep onset
